# Integrated genomics and functional validation identifies malignant cell specific dependencies in triple negative breast cancer

**DOI:** 10.1038/s41467-018-03283-z

**Published:** 2018-03-13

**Authors:** Nirmesh Patel, Daniel Weekes, Konstantinos Drosopoulos, Patrycja Gazinska, Elodie Noel, Mamun Rashid, Hasan Mirza, Jelmar Quist, Fara Brasó-Maristany, Sumi Mathew, Riccardo Ferro, Ana Mendes Pereira, Cynthia Prince, Farzana Noor, Erika Francesch-Domenech, Rebecca Marlow, Emanuele de Rinaldis, Anita Grigoriadis, Spiros Linardopoulos, Pierfrancesco Marra, Andrew N. J. Tutt

**Affiliations:** 1 Breast Cancer Now Research Unit, King’s College London, London, SE1 9RT UK; 2 School of Cancer and Pharmaceutical Sciences, King’s Health Partners AHSC, Faculty of Life Sciences and Medicine, King’s College London, London, WC2R 2LS UK; 3 The Breast Cancer Now Toby Robins Research Centre, The Institute of Cancer Research, London, SW7 3RP UK; 4 Cancer Bioinformatics, King’s College London, London, SE1 9RT UK; 5 Precision Immunology Cluster, Sanofi, 640 Memorial Drive, Cambridge, MA 02149 USA; 6 Cancer Research UK Cancer Therapeutics Unit, The Institute of Cancer Research, London, SM2 5NG UK

## Abstract

Triple negative breast cancers (TNBCs) lack recurrent targetable driver mutations but demonstrate frequent copy number aberrations (CNAs). Here, we describe an integrative genomic and RNAi-based approach that identifies and validates gene addictions in TNBCs. CNAs and gene expression alterations are integrated and genes scored for pre-specified target features revealing 130 candidate genes. We test functional dependence on each of these genes using RNAi in breast cancer and non-malignant cells, validating malignant cell selective dependence upon 37 of 130 genes. Further analysis reveals a cluster of 13 TNBC addiction genes frequently co-upregulated that includes genes regulating cell cycle checkpoints, DNA damage response, and malignant cell selective mitotic genes. We validate the mechanism of addiction to a potential drug target: the mitotic kinesin family member C1 (KIFC1/HSET), essential for successful bipolar division of centrosome-amplified malignant cells and develop a potential selection biomarker to identify patients with tumors exhibiting centrosome amplification.

## Abstract (web summary)

Triple negative breast cancers harbor multiple copy number aberrations driving gene expression changes thought to underpin their malignant phenotypes. Here the authors integrate these features, finding and functionally validating 37 gene addictions among which they identify the mechanism of addiction to KIFC1, a potential selective drug target.

## Introduction

Triple negative breast cancers (TNBCs) are difficult to treat and lack expression of the validated breast cancer therapeutic targets: estrogen (ER), progesterone (PR), and human epidermal growth factor 2 (HER2) receptors^1^. TNBCs are heterogeneous^2^ with substantial numbers of patients in subgroups that have high risk of early metastatic relapse commonly resistant to systemic therapy. Despite frequent resistance, chemotherapy is the only widely accepted systemic therapy option for these patients, highlighting the need to better understand the underlying biology and identify tumor cell-specific therapy targets for drug discovery or “repositioning” of known therapies.

Identification of tumor addictions (dependence on a gene for proliferation and survival) has in the past led to the development of novel therapies, notably the discovery of *ERBB2* amplification and overexpression, now targeted by a number of therapies in breast cancer^3^. Despite progress in characterizing the genomic landscape of breast cancer^4,5^ and TNBC specifically^2,6–8^, targetable biological dependencies remain elusive and poorly characterized. With the exception of clonally dominant mutations in *TP53*, TNBCs demonstrate a high degree of inter-tumor and intra-tumor heterogeneity at the mutational level with each driver mutation only present in a subset of tumors and clones within any individual tumor^9^.

TNBCs have a high frequency of chromosomal instability resulting in variable copy number state and levels of gene expression^7,10^. Genes that are found in amplified regions and are highly expressed, may be drivers of important “hallmarks” of malignancy^11^ and potentially represent essential tumor addictions. A number of high-throughput loss of function screening studies have identified gene addictions in cellular models of cancer including breast cancer models^4,5,12^ but functional validation has been limited and studies have rarely been informed by evidence of upregulation of gene copy number or mRNA in large numbers of patient tumors. Therefore, the main aim of this study is to identify and validate recurrently amplified genes as being important for malignant phenotypes in TNBC. We perform a pre-specified integrative computational “driver” identification and RNAi-based functional validation approach, taking into account both the copy number landscape and whole genome expression state in individual tumors, using a large discovery cohort of TNBCs. We further couple this with clinical, functional and “druggability” annotation to identify, cross reference in external data sets, and then functionally validate, potential tumor addictions in TNBC.

As expected this approach identifies both known and novel genes that are required for the survival of TNBCs. Interestingly, we identify clusters of genes that are more frequently co-upregulated in TNBC. Within the largest cluster, recurrent across external data sets, we focus on a potential drug target the kinesin family member C1 (KIFC1/HSET) and show that KIFC1 is a selective essential gene for many malignant breast cancer cells, demonstrating the mechanism of addiction to be based upon clustering of abnormal multiple centrosomes relevant to the majority of TNBCs that have centrosome amplification^13^. Furthermore, we developed a potential centrosome abnormality biomarker applicable to routinely fixed paraffin-embedded tumor tissue to enable patient segmentation of those with cancers susceptible to KIFC1/centrosome amplification targeted therapy.

## Results

### Identification of candidate gene addictions

**Fig. 1:**
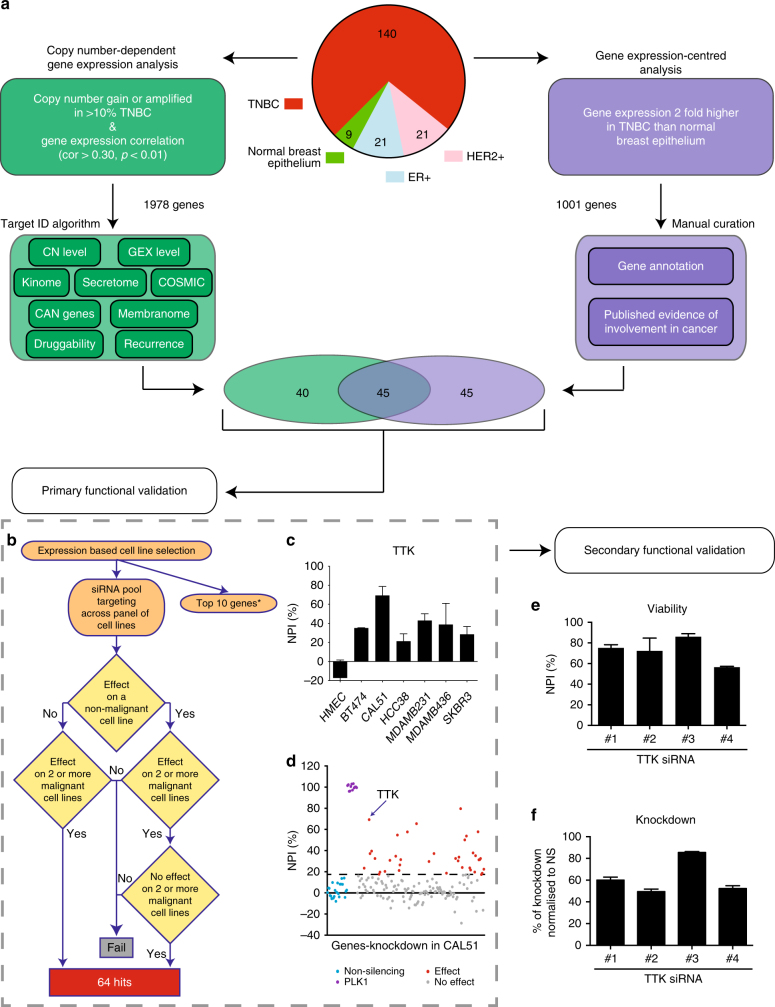
Integrative gene addiction identification and validation. Schematic representation of bioinformatics based “Target ID” and functional validation RNAi experiment. **a** Composition of the Guy’s TNBC-enriched cohort of breast cancers, used as a source of DNA and RNA for this study. Two complementary approaches for candidate gene addiction identification. In green, copy number-dependent gene expression analysis shows initial filter for copy number gain/amplification and correlation to gene expression and subsequent Target ID algorithm. This pipeline consisted of a weighted scoring system for all genes based on copy number (CN), gene expression (GEX), gene, and clinical annotation listed here and in the Methods section. In purple, gene expression-centered analysis followed by manual curation based on gene annotation and literature evidence of an involvement in malignancy. **b** Workflow and hit selection criteria from primary functional validation RNAi experiment. *Top 10 genes were subject to different criteria as outlined in Results and Methods. **c** Example functional validation for TTK, using pool of four siRNAs across panel of cell lines. Mean normalized percent inhibition (NPI) from three independent experiments is plotted and error bars represent the standard error of the mean (SEM), *n* = 3. **d** Example of data from primary functional validation carried out in CAL51 cells. Data points represent the mean NPI from three independent replicates. Dashed line represents cut-off for positive hits at 18.01% NPI. **e** Mean NPI from CAL51 cell line after deconvolution of pool of TTK siRNAs used independently. Error bars represent SEM, *n* = 3. **f** mRNA knockdown of individual siRNAs in CAL51 cells. Knockdown was evaluated by qPCR and represented as mean percentage of knockdown compared to non-silencing control. Error bars represent SEM, *n* = 3

In order to identify candidate tumor addiction genes, we interrogated the genome-wide Affymetrix SNP6.0 copy number and Human Exon 1.0ST gene expression profiles of 140 TNBC, 21 HER2-positive/ER-negative and 21 HER2-negative/ER-positive breast cancers, and 9 normal breast epithelium samples^14–16^. All clinico-pathological features of the cohort are provided in Supplementary Data 1. We obtained gene-centric copy number levels, frequency and focality of gene copy number changes as well as the gene expression from this cohort (Fig. 1a). The data were integrated with analyses of publicly available databases such as COSMIC^17^, the membranome^18^, the druggable genome^19^, secretome^20^, CAN genes^21^, and kinome^22^ (Supplementary Data 2). All the above data were collated in the Target ID data platform that was used as a foundation for the application of a pre-specified selection algorithm for putative addiction genes in TNBC.

The Target ID data platform informed two complementary approaches to gene selection for functional validation (mRNA overexpression and gene amplification/ mRNA expression correlation) with the aim being to minimize bias and limitations inherent to any single analytical procedure. First, a copy number-dependent gene expression analysis selected 1978 candidate genes amplified in >10% of TNBCs with a gene copy number/gene expression correlation (*r*^2^ > 0.3, *p* < 0.01, Spearman’s rank correlation). We then linked the candidate genes to features, included in the Target ID data platform, which are known to be relevant to hallmarks of malignancy in a weighted scoring system (Fig. 1a and Supplementary Data 3). The top 85 genes were taken forward for functional validation.

Second, a complementary gene expression-centered analysis was used as there is evidence that variations in the expression of tumor addiction genes may also occur in the absence of in cis CNAs through multiple mechanisms, for example through epigenetic regulation^23^. For the gene expression-centered analysis, we identified 1001 genes whose average expression in our TNBC cohort was >2-fold higher than normal breast epithelium controls. As anticipated by the inclusion of CNA correlated elevated gene expression in the first approach there was a substantial overlap with 45 of the genes from the second approach which were taken forward for functional validation. An additional 45 genes identified by this second approach were selected based exclusively on gene annotation and literature review. This manually curated filtering sought to identify genes already shown to drive a tumor phenotype in other cancer models or being involved in biological pathways known to be key in cancer development and progression. In total our approaches identified 130 candidate tumor addiction genes (Fig. 1a) with a high degree of overlap with both TCGA^6^ and METABRIC^7^ data sets and (Supplementary Datas 4 and 5).

### Functional validation of candidate genes using RNAi

To assess the role of each of the 130 candidate gene addictions in the proliferation and viability of cancer cells, siRNA knockdown and cell viability assays were carried out in breast cancer cell lines(BCCLs). In order to increase the feasibility of the initial candidate screening experiment, each gene was silenced in a gene-specific subset of 6–9 BCCLs (out of a panel of 16) and the non-malignant HMEC normal breast epithelial cell line. Expression levels of each gene were analyzed in our cell line cohort based on previously described mRNA analyses^14,24^. Cell lines were selected for each gene to capture the widest possible range of expression levels of each gene (Supplementary Figure 1 and Supplementary Data 6).

As candidate genes were assessed across multiple cell models, normalized percent inhibition (NPI) of growth was used to compare data obtained from multiple experiments with different cell lines. Repeated measures of the NPI of negative controls across all cell lines showed a mean of 0.18% with a standard deviation of 5.94 (Supplementary Figure 2a). Therefore, the knockdown of a gene was considered as having a growth inhibition effect on a cell line, if its knockdown achieved an NPI ≥ 18.01% (three standard deviations above the mean).

Based on highest scores from the Target ID algorithm, literature review and expert opinion a group of 10 genes amongst the 130 candidates (Top 10—see Fig. 1b) were selected as being more likely to be associated with a malignant cell-specific addiction in TNBC and were expedited for multiple single oligo RNAi analysis of effect on phenotype with validation of gene knockdown from the outset. For these genes, a prioritized validation procedure was employed which involved independent testing of three individual siRNAs per gene for evidence of gene knockdown by qPCR in relation to phenotypic effect in a proliferation assay. We aimed to identify genes that, rather than being essential for the viability of all cells, showed growth inhibition effect on some but not all cancer cells indicating some selectivity for an underlying biological context and malignant phenotype. Therefore, genes from this “top 10” group were considered a “hit”, if two siRNAs that showed a knockdown >70% did not affect the growth of HMEC, but did inhibit the growth of at least two cancer cell lines. We identified seven genes (*FZD6*, *MASTL*, *NCSTN*, *PTK2*, *PTP4A3*, *SEC61G*, and *UBE2T*) from this group as being putative tumor addiction genes (Supplementary Datas 4 and 7).

The remaining 120 genes identified through the integrated analyses underwent a two-stage functional validation process. First, each gene was assessed using a pool of four siRNAs. A gene was considered a “hit” if its silencing inhibited growth in at least two cancer cell lines whilst having no effect on HMEC. By example, we declared *TTK/MPS1*, a previously validated mitotic target^25^, as a “hit” by these criteria in the screen (Fig. 1b–d). As HMEC themselves may have developed dependencies as a result of “in vitro” 2D culture on plastic, we also considered a gene a “hit” if it showed an effect on HMECs, but had a heterogeneous effect across the cell line panel with no effect in at least two cancer cell lines indicating that the gene was not essential for all dividing cells. Primary validation revealed 64 potential tumor addiction genes (Supplementary Datas 4 and 8 and Supplementary Figure 2b).

To identify and discard false positive “hits” that were in fact due to off-target effects within our RNAi pools, we performed a secondary functional validation step. This required there to be consistency between relative mRNA knockdown and cell viability changes for each of the four siRNAs used independently (selection criteria are described in detail in Online Methods) (Fig. 1e,f). Of the 64 genes, 30 fully met these secondary functional validation criteria, and 34 failed (Supplementary Datas 4 and 9).

Overall, the primary and secondary RNAi-based functional analyses have validated 37 tumor addiction genes (7 from the “top 10” and 30 of the remainder (Supplementary Figure 3 and Table 1.) All “out of patient” functional validation models have their caveats. The stress of cell culture may change relationships between gene expression and CNAs and cell addiction in comparison to that in the patient. Although our identification of these genes in genomics data was based on integrated copy number and gene expression with evidence of overexpression or CNA-driven expression in patient primary tumors, we believe that a requirement for linkage of amplification or level of gene expression with phenotype in 2D cell cultures would be a poor validation criterion for identifying selective dependencies. Rather we require heterogeneity of siRNA effect on a gene across multiple cell lines, including a non-malignant model, followed by validation of linkage between phenotype and mRNA depletion across multiple siRNAs for these 37 genes believing this to be a better criterion to find selective tumor addictions, as opposed to non-selective obligate requirements for a gene for cell growth in all cells. The targeting of the latter gene would be expected to create adverse effect in normal tissues in patients. Despite the caveats raised above, for completeness, we show the depletion induced phenotype of each validated gene in each cell line used showing that cell line’s expression of the gene relative to transformed but non-malignant HMEC cells. We found 10 out of the 37 genes selected based on our criteria also had evidence of gene dependency only in cell lines with levels of gene expression greater than that of HMECs (Supplementary Figure 4).

**Table 1:** Functional annotation of validated genes

	

The 37 functionally validated genes and their manually curated gene annotation of biological function using the GeneCards Suite^26^. Red, tumor gene addictions novel to cancer, orange, tumor gene addictions novel to breast cancer

### Identification of functional clusters required by TNBC

**Fig. 2:**
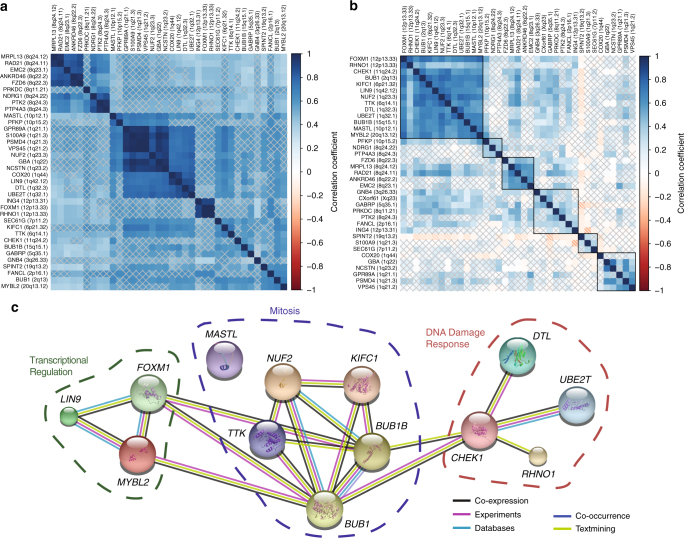
A subset of tumor addiction genes that are co-upregulated have roles in cell cycle progression, mitosis and DNA damage response. Copy number (**a**) and gene expression (**b**) levels of 37 tumor addiction genes were pairwise correlated and tested for statistical significance using Pearson method in the TNBC tumors of the Guy’s TNBC-enriched cohort (*n* = 82). Their correlation coefficients were hierarchically clustered using Ward distance method, and displayed as levelplots. In the gene expression correlation heatmap highly correlated genes are surrounded by black rectangles, representative of underlying clusters from hierarchical clustering method whereas a cross denotes insignificant *p*-value as per level of 0.05. **c** Interconnectivity among the 13 co-upregulated tumor addiction genes is displayed based on STRING networks. Genes with shared functional roles are outlined

Literature review, the GeneCards database^26^ and gene annotation analyses of these 37 genes revealed involvement in the cellular processes of cell cycle regulation, DNA damage response, epigenetic regulation, metabolism, proteasome function, protein sorting, signaling, and vesicle trafficking (Table 1). To understand coordinated upregulation that might drive the biology of TNBCs we interrogated the pattern of amplification/upregulation of these genes, across our tumor cohort and, for external validation, in public databases. Among the 37 genes, we found as expected genes that frequently had their copy number levels correlated when they reside in close proximity in the genome (Fig. 2a). We sought to identify co-upregulation of genes which are not in the same amplicon or in close vicinity and so performed pairwise gene to gene expression correlation, through which a set of 13 highly co-upregulated genes was identified (Fig. 2b). Using the expression levels of these 13 genes to create a composite score clearly identified a population of ~88% of TNBC patients with a high expression of this score (Supplementary Figure 5a, b). This high scoring population contains all the basal-like 1, basal-like 2, and immunomodulatory Vanderbilt TNBC subtype^2^ tumors but does not exclude other types (Supplementary Figure 5c). These 13 genes are involved in the regulation of the transcription of cell cycle progression genes (*FOXM1*, *LIN9*, and *MYBL2*)^27^, in the DNA damage response pathway (*CHEK1*, *DTL*, *RHNO1*, and *UBE2T*)^28,29^, and in mitosis (Fig. 2c). Among these malignant cell selective addiction genes associated with mitosis *MASTL* regulates mitotic entry^30^, *BUB1*, *BUB1B*, *NUF2*, and *TTK* act as part of the spindle assembly checkpoint^31^, and *KIFC1* has been shown to play an essential role in centrosome clustering to regulate bipolarity during mitosis^32^. These data were supported by analysis of publicly available data sets (Supplementary Figure 5d–g) where we found 10 genes (*BUB1*, *CHEK1*, *DTL*, *FOXM1*, *KIFC1*, *LIN9*, *NUF2*, *RHNO1*, *TTK*, and *UBE2T*) concordant between analyses of our data and in the METABRIC study dataset. Moreover, when we investigated the 5′ upstream regions of these genes and identified an *E2F8* transcription factor binding site in 8 out of 13 genes, namely *KIFC1*, *MYBL2*, *TTK*, *CHEK1*, *FOXM1*, *NUF2*, *UBE2T*, and *MASTL* (Supplementary Figure 5h). Expression levels of *E2F8* were highly correlated with each of the eight genes and might point to a common transcriptional activation network that further enhances the copy number-dependent expression of these genes.

Although many cancer therapeutics target the mitotic apparatus, the ability to selectively impact mitosis in malignant cells has largely evaded drug discovery efforts. There is evidence that KIFC1, a potentially druggable ATP-dependent motor protein^33^, is involved in tumorigenesis through its ability to cluster extra centrosomes in centrosome-amplified cancer cells. In addition, centrosome amplification is a major cause of aneuploidy and genomic instability^34^ all of which are highly prevalent in breast cancers^35^. Therefore, we focused on the validation of KIFC1 CNAs and expression in external data sets and the further investigation of its function in the malignant cell-specific dependency in TNBC.

**Fig. 3:**
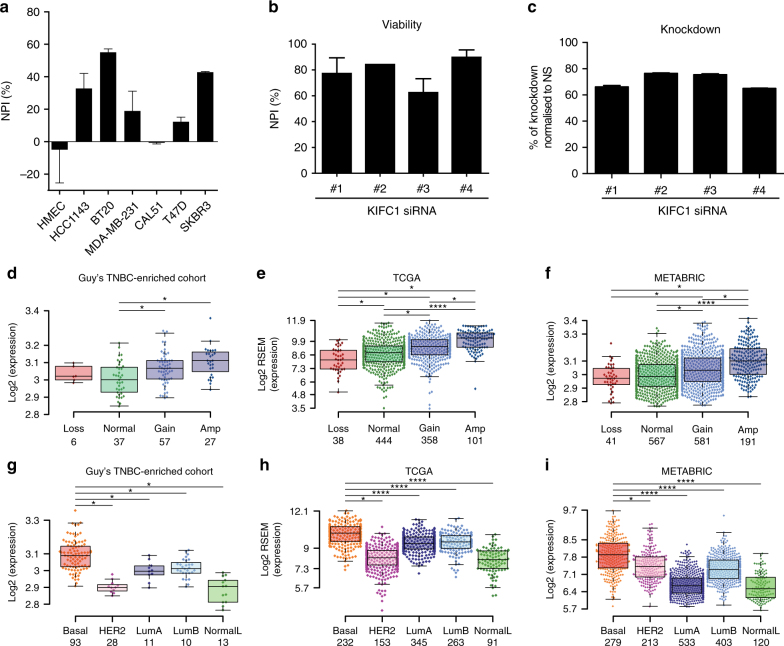
KIFC1 is a validated tumor addiction gene that is upregulated in TNBCs. **a** Primary pooled siRNA oligo validation data for KIFC1. Mean NPI are plotted and error bars represent the SEM, *n* = 3. **b** Mean NPI from three independent of HCC1143 cell line after deconvolution of pool of KIFC1 siRNAs used independently. Error bars represent SEM, *n* = 3. **c** mRNA knockdown of individual siRNAs in HCC1143 cells. Knockdown was evaluated by qPCR and represented as percentage of knockdown compared to non-silencing control from three technical replicates. **d** Copy number alterations of *KIFC1* correlated with its gene expression across all breast cancers in the Guy’s TNBC-enriched cohort of breast cancers, (**e**) TCGA BRCA and (**f**) METABRIC data sets. **g** *KIFC1* gene expression across the PAM50 breast cancer subtypes in the Guy’s TNBC-enriched cohort of breast cancers, (**h**) TCGA BRCA and (**i**) METABRIC data sets. Box-and-whisker plots showing median center line, 25% and 75% box limits and range of expression, non-paired two-sided Wilcoxon rank sum test; *p < 0.05, ****p < 0.0001

The heterogeneous effect of silencing *KIFC1* across the panel of seven cell lines used for its primary and secondary functional validation (Fig. 3a), suggests a mechanism-specific dependency rather than simply a requirement for this kinesin motor protein in all highly proliferative cells. Our secondary functional validation by deconvolution of the siRNA pool, with demonstration of effect of all four siRNAs in the pool and proof of knockdown, reduce the likelihood the phenotype is caused by an off-target effect of an siRNA (Fig. 3b, c).

We next sought confirmation of the relationship between KIFC1 CNA and gene expression in external and independent TCGA Breast and METABRIC data sets^6,7^ and observed a direct correlation between *KIFC1* gene expression and gene copy number similar to that seen in our own discovery cohort (Fig. 3d–f). In addition, to investigate if *KIFC1* expression is breast cancer subtype specific, its expression levels were analyzed across PAM50 breast cancer subtypes, demonstrating higher levels of *KIFC1* in the basal-like subtype, known to have significant overlap with, and forming the dominant subtype within, TNBC (Fig. 3g–i).

### Centrosome amplification sensitizes cells to KIFC1 silencing

**Fig. 4:**
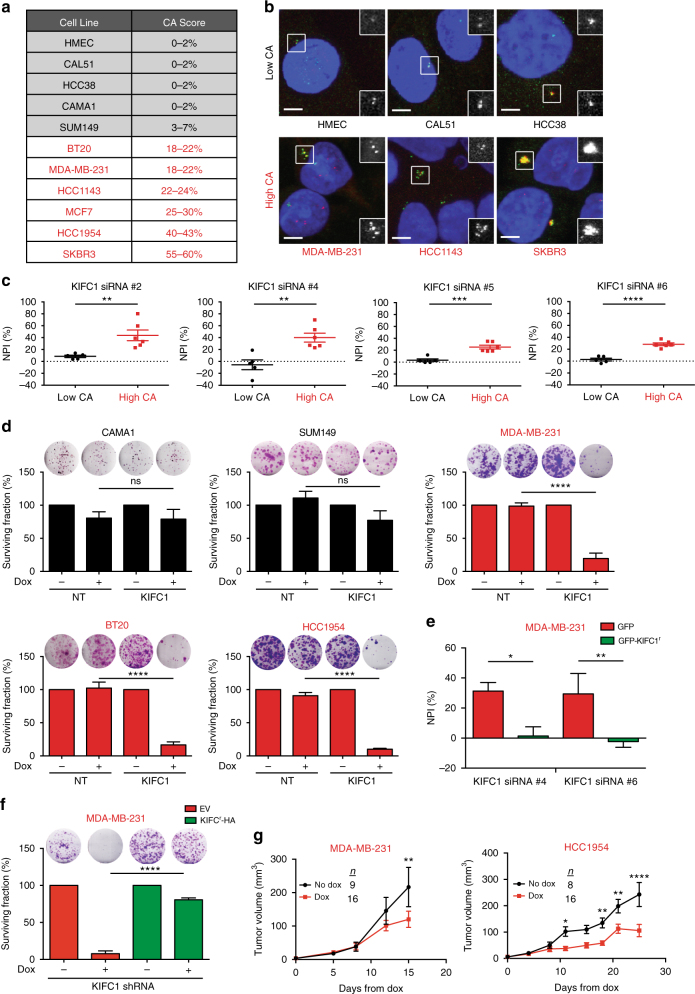
KIFC1 is specifically required for survival of cancer cells exhibiting centrosome amplification. **a** Centrosome amplification (CA) scores for panel of breast cancer cell lines. Cell lines were dichotomized into low CA (black) and high-CA (red) groups. **b** Representative immunofluorescence images showing centrosome marker Aurora A (green) and centriole marker CP110 (red) used for calculating CA score. Scale bar is 5 μm. Top right inset shows red channel, bottom right inset for each image shows green channel. **c** Mean normalized percent inhibition (NPI) of panel of cell lines dichotomized into low and high-CA groups for siRNA #2, #4, #5, and #6. Error bars represent the SEM, *n* > 3. Student's *t* test: **p < 0.01, ***p < 0.001, ****p < 0.0001. **d** Colony formation assay of two low centrosome-amplified cell line (CAMA1 and SUM149) and two high-centrosome-amplified cell lines (MDA-MB-231, HCC1954 and BT20) infected with either non-targeting shRNA (NT) or shRNA-targeting KIFC1 (KIFC1 shRNA). Cells were grown for 14–21 days in the presence or absence of doxycycline, fixed and stained in crystal violet and colonies quantified. Mean surviving fraction normalized to NT, error bars represent the SEM, *n* = 3. One-way ANOVA with Tukey’s multiple comparisons test: ***p < 0.001, ****p < 0.0001. **e** NPI of MDA-MB-231 cells infected with either GFP alone (GFP) or GFP-tagged RNAi-resistant KIFC1 (GFP-KIFC1^r^) with KIFC1 siRNA #4 and #6. Error bars represent the SEM, *n* = 3, one-way ANOVA with Tukey’s multiple comparisons test: *p < 0.05, **p < 0.01. **f** Colony formation assay of MDA-MB-231 cells infected with inducible KIFC1 shRNA and infected with inducible RNAi-resistant HA-tagged KIFC1 (KIFC1-HA^r^) or with empty vector (EV) control. Mean surviving fraction normalized to NT, error bars represent SEM, *n* = 3. One-way ANOVA with Tukey’s multiple comparisons test: ****p < 0.0001. **g** Nude hosts were orthotopically injected with either MDA-MB-231 or HCC1954 cells with inducible KIFC1 shRNA and were treated with (red) or without (black) doxycycline when tumors reached >2 × 2 mm (4.2 mm^3^). Mean tumor volumes at time points indicated, error bars represent the SEM, from two independent experiments. Two-way ANOVA with Sidak’s multiple comparisons test: *p < 0.05, **p < 0.01, ****p < 0.0001

KIFC1 has been shown to play a role in centrosome clustering, generation and maintenance of bipolar mitosis in cells exhibiting supernumerary centrosomes^32,36^. To determine whether the dependency of our breast cancer models on KIFC1 was related to the degree of centrosome abnormality they demonstrate, 11 cell lines were scored for the proportion of cells with centrosome amplification and subsequently tested for functional dependency on KIFC1 by using RNAi. CAL51, HCC38, CAMA1, SUM149, and the non-malignant breast cell line, HMEC, had low levels of centrosome amplification (0–7%), while BT20, MDA-MB-231, MCF-7, HCC1143, HCC1954, and SKBR3 showed relatively high levels of centrosome amplification (18–60%) (Fig. 4a, b).

Four independent siRNA oligos, validated to deplete KIFC1 mRNA and/or protein (Supplementary Figure 6a, b), induced a substantial reduction of cell viability over a 6-day period only in the cell models with centrosome amplification, regardless of breast cancer subtype (Fig. 4c). *KIFC1* knockdown showed an even greater impact in a long-term clonogenic survival assay (14–21 days) in centrosome-amplified cell lines, MDA-MB-231, HCC1954, and BT20 using an independent shRNA sequence targeting *KIFC1* in an inducible expression system (Fig. 4d; Supplementary Figures 6c, 9). In contrast, no significant reduction in clonogenic survival was seen in the CAMA1 cell line, which has low levels of centrosome amplification (one-way ANOVA with Tukey’s multiple comparisons test).

To further exclude the possibility that, despite using multiple RNAi approaches, the observed phenotype was due to an “off target” effect, we performed a rescue of function experiment by over-expressing an RNAi-resistant GFP-tagged or a HA-tagged KIFC1 protein (GFP-KIFC1^r^/KIFC1^r^-HA) in MDA-MB-231 cells. As shown in Fig. 4e, reduction in cell viability by KIFC1 siRNA knockdown was rescued by the presence of GFP-KIFC1^r^ (Supplementary Figure 6d). Similarly, the long-term clonogenic survival phenotype was rescued in the presence of KIFC1^r^-HA following knockdown with shRNA (Fig. 4f; Supplementary Figure 6e), confirming the specificity of the dependency on KIFC1.

We subsequently examined the consequence of *KIFC1* silencing on the tumor growth of centrosome-amplified cell line xenografts with MDA-MB-231 and HCC1954 cells in vivo. Using an inducible shRNA expression system with *KIFC1* shRNA doxycycline-induced KIFC1 depletion resulted in significant tumor growth inhibition in both cell lines (Fig. 4g) in contrast to non-targeting (NT) control cell xenografts (Supplementary Figure 7a) (two-way ANOVA with Sidak’s multiple comparisons test). KIFC1 depletion in tumors was confirmed by immunohistochemistry (IHC; Supplementary Figure 7b). We hypothesized that the ability of mitotic cells to cluster supernumerary centrosomes into a bipolar mitosis in xenograft tumors would be impaired by KIFC1 silencing. Histological analysis of centrosomes by pericentrin (an integral component of the pericentriolar material) by IHC and identification of mitotic cells by nuclear counter staining (Supplementary Figure 7c), confirmed that loss of KIFC1 resulted in a reduction in the proportion of mitoses in centrosome-amplified cells where cells were capable of clustering their centrosomes into two poles (bipolar), 4 days after the start of treatment in both cell lines (Supplementary Figure 7d).

### KIFC1 silencing causes multicentrosome multipolar mitosis

**Fig. 5:**
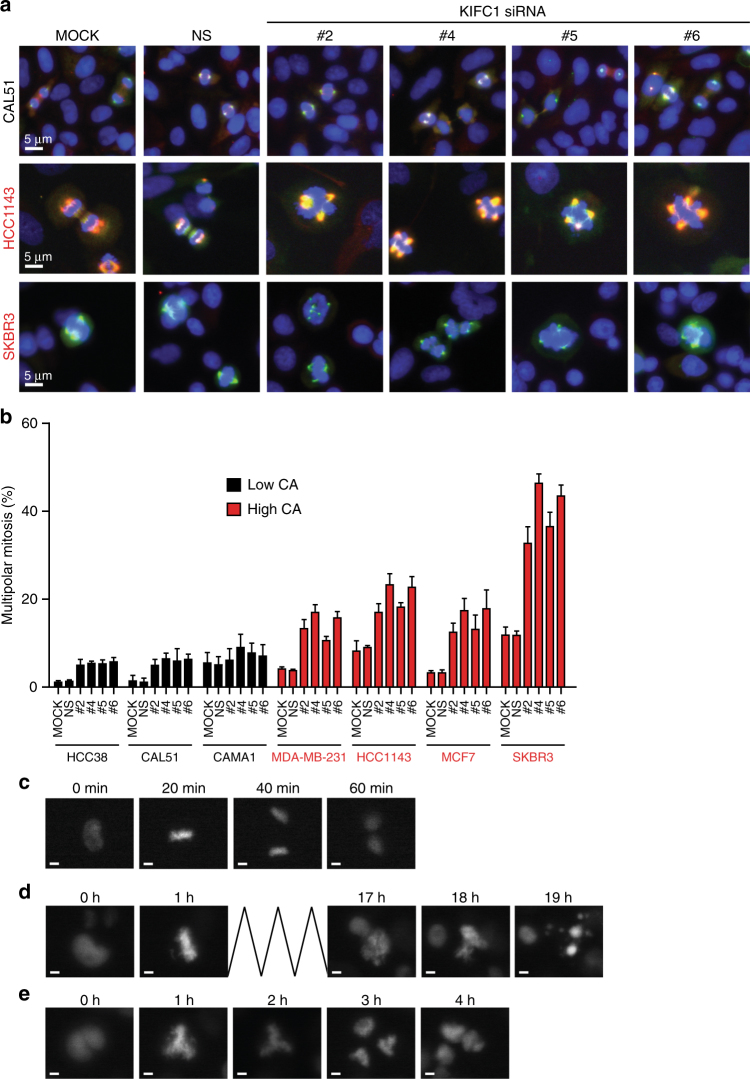
Cell lines with centrosome amplification undergo multipolar mitoses followed by mitotic catastrophe in the absence of KIFC1. **a** Representative immunofluorescence images of mitotic spindles in low-CA cell line (CAL51) and high-CA cell lines (HCC1143 and SKBR3) with control or KIFC1 siRNA or mock transfection. Spindles stained with Aurora A (green) and/or Eg5 (red). Scale bar is 5μm. **b** Mean percentage of multipolar mitotic cells. Error bars represent the SEM, *n* = 3. **c** Images of time-lapse of MDA-MB-231 cells expressing NT shRNA showing a bipolar mitosis. **d** Images of time-lapse of MDA-MB-231 expressing KIFC1 shRNA showing prolonged mitosis and subsequent apoptosis. **e**. Images of MDA-MB-231 cells with inducible KIFC1 shRNA showing an abnormal multipolar mitosis. Scale bar is 5 μm

To investigate the mechanism by which centrosome-amplified breast cancer cells are “addicted” to KIFC1, comparative immunofluorescence imaging of the mitotic spindle was performed in three cell lines with low levels of centrosome amplification and five cell lines with relatively high levels of centrosome amplification. There was a marked increase in the number of mitotic spindle poles per cell and consequent multipolar mitoses in the cell population when KIFC1 was knocked down in the centrosome-amplified cells (Fig. 5a, b).

Next, using live cell time-lapse imaging of MDA-MB-231 cells, we investigated the fate of cells undergoing multipolar mitosis during knockdown of *KIFC1*. We demonstrated that *KIFC1* knockdown arrested cells in mitosis for a prolonged period, compared to a normal bipolar mitosis (Fig. 5c; Supplementary Movie 1), and consequently caused the cells to either undergo mitotic catastrophe (Fig. 5d; Supplementary Movie 2) or to fail cytokinesis and remain in a multinucleated state (Fig. 5e; Supplementary Movie 3). The druggability of KIFC1 has previously been shown where the KIFC1 inhibition tool compound, AZ82, is predicted to contact the L5 loop in an ATP competitive manner and showed a signal of efficacy in centrosome declustering but had poor target potency and had many off-target non-centrosome amplification selective cytotoxic effects^33^. We have confirmed this (Supplementary Figure 8) precluding its further use in our work but raising the possibility of discovery of more potent and specific KIFC1 kinesin motor inhibitors.

### Induced centrosome amplification increases KIFC1 dependency

**Fig. 6:**
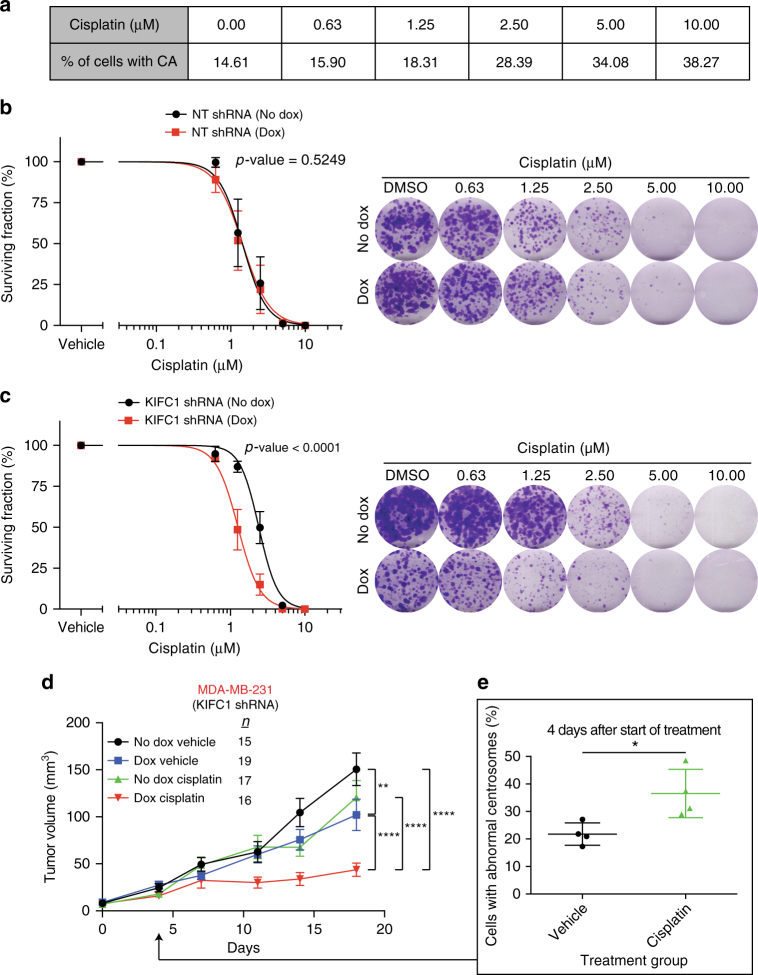
Therapeutic induction of centrosome amplification in tumors increases the efficacy of KIFC1 silencing. **a** MDA-MB-231 cells were treated with five serial concentrations of cisplatin and DMSO control and scored for centrosome amplification (CA). Table shows the level of CA at each concentration of cisplatin. **b** Surviving fraction of MDA-MB-231 cells with inducible NT shRNA with (red) or without (black) doxycycline. Data points represent the mean, error bars represent the SEM, *n* = 3. Extra sum-of-square *F*-test (*F*-value 0.41). Right, representative images of colony formation wells for each treatment condition. **c** Surviving fraction of MDA-MB-231 cells with inducible KIFC1 shRNA with (red) or without (black) doxycycline. Data points represent the mean, error bars represent the SEM, *n* = 3. Extra sum-of-squares *F*-test (*F*-value 57.59). Right, representative images of colony formation wells for each treatment condition. Note, five times more MDA-MB-231 cells with KIFC1 shRNA (Dox) were plated due to low colony forming ability of cells without KIFC1. **d** Nude hosts were injected with MDA-MB-231 cells with inducible KIFC1 shRNA and were treated with or without doxycycline and either vehicle or 5 mg kg^−1^ of cisplatin when tumors reached >2 × 2 mm or 4.2 mm^3^. Mean tumor volume at time points indicated, error bars represent the SEM, from two independent experiments. Two-way ANOVA with Tukey’s multiple comparison test: **p < 0.01, ****p < 0.0001 (only shown for final time point). **e** Nude hosts were injected with MDA-MB-231 cells with inducible KIFC1 shRNA and were treated with either vehicle or 5 mg kg^−1^ of cisplatin when tumors reached >33 mm^3^. Tumors taken 4 days after start of treatment in the no dox group were stained with pericentrin by IHC and the percentage of abnormal centrosomes was scored for vehicle and cisplatin treatments arms. Mean percentage of cells with centrosome abnormalities, error bars represent the SEM, *n* = 4. Student's *t* test: *p < 0.05

KIFC1 depletion in vivo resulted in a clear reduction in tumor growth but not total inhibition of growth. As there is no specific and potent KIFC1 motor inhibitor we used inducible shRNA interference to deplete KIFC1 expression. Using RNAi, continued function of a small amount of residual protein is likely to limit efficacy in comparison to a drug. In addition, non-centrosome-amplified sub-populations of cells may be unaffected and persist leading to a growth impaired but viable tumor. We therefore sought to augment the effect of KIFC1 depletion by combining it with a clinically relevant therapy capable of further induction of centrosome amplification in tumor cells. Platinum-based chemotherapies are currently considered one of the standard of care treatments for advanced TNBCs^37^ and cisplatin is known to cause centrosome amplification by decoupling the centrosome duplication cycle from the DNA replication cycle^38^. We found that MDA-MB-231 cells show an increase in centrosome amplification when treated with increasing concentrations of cisplatin (Fig. 6a). We therefore hypothesized cisplatin would increase cellular dependency on KIFC1 and by normalizing colony formation assays to vehicle control in the presence or absence of doxycycline, we show synergy beyond additivity between the effect of cisplatin treatment and inducible shRNA-induced KIFC1 depletion (Fig. 6b, c) consistent with the increase in centrosome amplification induced by cisplatin.

We investigated the combination of cisplatin treatment and KIFC1 silencing in MDA-MB-231 cell line xenografts again using an inducible shRNA expression system. Cisplatin treatment led to sensitization to KIFC1 silencing compared to cisplatin or KIFC1 silencing alone (Fig. 6d) consistent with the synergistic effect seen in vitro in the same model. Pericentrin staining of MDA-MB-231 xenografts after 4 days of cisplatin treatment revealed significantly increased centrosome abnormalities as compared to vehicle treated tumors in the absence of doxycycline (Fig. 6e) (Student's *t* test).

### A potential biomarker for tumor addiction to KIFC1 function

**Fig. 7:**
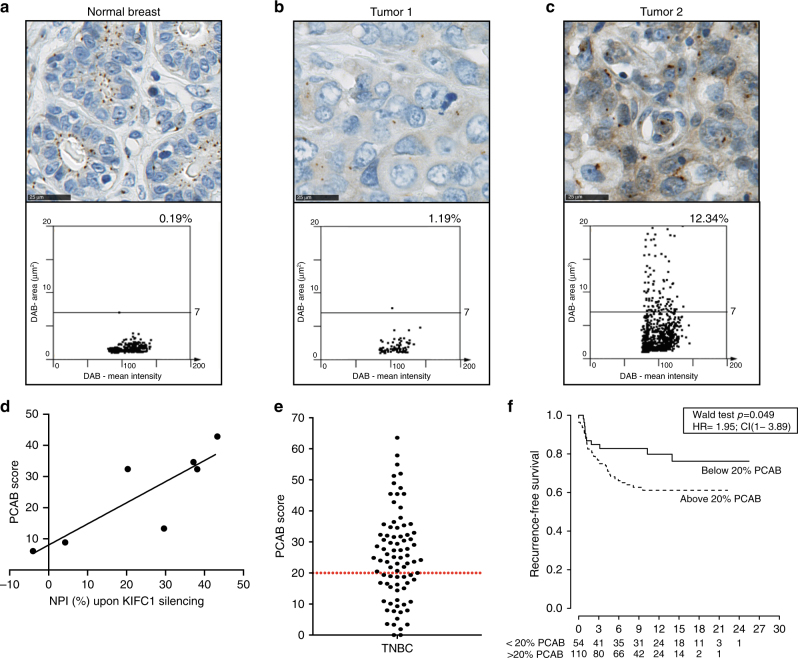
Pericentrin abnormality score: a potential predictive biomarker for sensitivity to KIFC1 inhibition. **a** Normal breast tissue section stained for pericentrin as a centrosome marker and below, a scatter graph of DAB staining area versus DAB mean intensity. Cut-off for normal centrosome size was set at 7 µm^2^. **b** Tumor 1 with centrosomes that appear normal. Below, scatter graph of DAB staining area vs DAB mean intensity showing a PCAB score of 1.19%. **c** Tumor 2 with centrosomes that appear abnormal. Below, scatter graph of DAB staining area vs DAB mean intensity showing a PCAB score of 12.34%. **d** PCAB score vs NPI (%) upon KIFC1 silencing across panel of breast cancer cell lines. Linear regression analysis, *r*^2^ = 0.71, *p* < 0.05. Scale bars represent 25 μm. **e**. PCAB score of TMAs of cohort of 82 TNBCs. Dashed red line depicts the median PCAB score (32%) of breast cancer cell lines sensitive to KIFC1. **f** Kaplan–Meier curves illustrating the duration of recurrence free survival according to a 20% PCAB cut-off. Wald test, *p* < 0.05, hazard ratio (HR) = 1.95, confidence intervals (CI) = 1–3.89

Use of immunofluorescence-based scoring of the percentage cells with centrosome amplification that we used in cell lines may be impractical as a predictive biomarker for KIFC1 motor silencing in patient formalin-fixed paraffin-embedded (FFPE) tumor material, because individual cells cannot be assessed throughout their volume and centrosomes are frequently lost outside the plane of the histological section. Centrosomes cannot be accurately counted and assigned to any individual cells. Therefore, we sought to develop a potential predictive biomarker based on identification of centrosome abnormalities in a light microscopy “field of view” using a pericentrin IHC assay. We developed an automated scoring system for analyzing the size of pericentrin stained centrosomes detected by IHC in interphase cells in FFPE tissue. Using image analysis, we sought to define the average size of a centrosome body in a normal breast tissue section (Fig. 7a) finding this to be 1.44 µm^2^ with the largest normal breast epithelial cell centrosome body being 7 µm^2^. We then set this as the cut-off for the upper limit of normal pericentrin stained centrosome size. A pericentrin staining body of greater size than this was termed “abnormal”. Based on this approach, we developed a pericentrin abnormality (PCAB) score, which we defined as the percentage of abnormal pericentrin stained bodies over total stained bodies in a whole section of FFPE-embedded cell line pellets or tumor sections. We subsequently compared centrosome body size and PCAB score in breast tumor sections that a pathologist determined demonstrated “normal” or “abnormal” centrosomes. We found that the former (Fig. 7b) had a centrosome body size and PCAB score in the range of that of normal breast tissue in contrast to the latter where our automated method correctly detected a higher PCAB score due to a number of “abnormal” pericentrin stained centrosome bodies within the field of view (Fig. 7c).

When the PCAB score was calculated in breast cancer cell line (BCCL) pellet FFPE blocks from lines previously characterized for KIFC1 dependency this revealed a linear correlation between PCAB score and the NPI caused by KIFC1 knockdown (Fig. 7d). In order to ascertain the proportion of TNBCs that might show centrosome amplification at a level we associate with KIFC1 dependency in vitro, automated PCAB scoring was performed on a panel of FFPE primary TNBCs. We found 63% of tumors had a PCAB score above 20%, above which no KIFC1-insensitive cell line was scored (Fig. 7e). This suggests that a substantial proportion of TNBCs have centrosome amplification at a level that may be associated with addiction to the centrosome clustering functions of KIFC1. The data also indicate patients with a high-PCAB score (>20%) had a shorter time to recurrence in the Guy’s TNBC-enriched cohort (Fig. 7f).

## Discussion

A number of comprehensive analyses have documented the genomic landscape of breast cancers and associated mutations, copy number variations and gene expression patterns but very few of these genomic features have been subjected to functional validation for their selective requirement for growth or other malignant phenotypes in breast cancer model systems^5,12^. TNBCs have few known targetable addictions^8,39^ and are dominated by copy number aberrations (CNAs) with in cis or in trans associated gene expression changes^7,10^. We have used a discovery cohort of triple negative enriched primary breast cancers and employed a pre-specified systematic integration of copy number and gene expression data within each tumor to identify and functionally validate genes, the expression of which are required for growth of cell models of breast cancer but not required by all proliferating cells.

Our analysis led to the identification of 37 functionally validated genes, (Table 1) many of which are novel, as well as confirmation of several known addictions in breast cancer that validate our approach.

Our findings identify a number of known oncogenes such as BUB1, BUB1B, and CHEK1 and less known and characterized gene addictions in TNBC such as FANCL, GABRP, GNB4, NUF2, and VPS45 which we find validated in other data sets^4,5^. Furthermore, comparison of validated hits and data from the COLT-Cancer data^12^ found concordance between our siRNA NPI scores and the zGARP shRNA scores for 11 genes (BUB1B, CHEK1, FOXM1, GABRP, MYBL2, PRKDC, PSMD4, S100A9, GPR89, FANCL, GNB4) (Supplementary Data 11). As expected given the differences in RNAi methodologies, phenotypic endpoint and lack of validation of knockdown effects of RNAi in the COLT-Cancer screen we observed little statistical correlation between the results overall^12^.

A correlation analysis showed several co-amplification and co-expression patterns across our tumors. Genes that were frequently co-amplified because of their association with the same amplicon, were not necessarily also co-upregulated. Interestingly 13 genes belonging to different amplicons and chromosomes showed a higher correlation in mRNA expression. Our data suggest that as well as being individually essential for TNBC survival, a substantial proportion of TNBCs rely on the function of specific genes within common cellular processes: such as the DNA damage response (*CHEK1*, *DTL*, *RHNO1*, and *UBE2T*); transcriptional regulation of the cell cycle as in the case of *FOXM1*, *LIN9*, and *MYBL2* which control the DREAM/LINC complex known to regulate entry/exit from quiescence and cancer cell proliferation^27^; and mitosis (*BUB1*, *BUB1B*, *KIFC1*, *MASTL*, *NUF2*, and *TTK*). Interestingly, *CHEK1*, *DTL*, and *MASTL* are implicated in the G2 checkpoint, while *NUF2*, *BUB1*, *TTK*, and *BUB1B* control the mitotic spindle assembly checkpoint.

In addition, this correlation analysis revealed cancer-specific addiction associated with coordinated upregulation of another subset of genes that reside in the same region of chromosome 1 and are involved in vesicle and protein trafficking (*GBA*, *GPR89A*, *NCSTN*, *PSMD4*, *VPS45*). Indeed, gene annotation analysis of the 37 functionally validated hits indicated others involved in vesicle and protein trafficking (*SEC61G*, *TTC35*, and *NRDG1*). Alteration of these processes have been suggested to be a causative event in cancer development^40^.

The largest of the clusters arising from the STRING protein interaction database network analysis identified malignant cell-specific dependencies on groups of mitosis and cell division control genes. Most basal-like cancers, which substantially overlap with TNBCs, exhibit high expression of proliferative genes and have a higher mitotic index than other breast cancer subtypes^41–43^. Importantly we find that knockdown of these genes is dispensable in non-malignant cells as well as some rapidly proliferating cancer models suggesting a specific requirement for the genes in these clusters due to selective addiction rather than an essential function in cell cycle progression and mitosis in all proliferating cells. Furthermore, the METABRIC study identified a common trans-acting chromosome 5q deletion that resulted in altered expression of signaling molecules and cell division genes in breast cancers of the intClust 10 subtype^7^. These genes include *AURKB, BUB1, CHEK1, FOXM1, KIFC1*, and *TTK*^7^, five of which, *BUB1, CHEK1, FOXM1, KIFC1*, and *TTK*, we have also identified and have functionally validated to have a selective requirement for cell growth in breast cancer cell models. The TTK/MPS1 kinase, is a druggable mitotic checkpoint kinase that has already been identified as a malignant cell selective target the depletion or inhibition of which causes failure of cell population growth associated with PTEN pathway deficiency^25^. The identification and functional validation of TTK/MPS1 is consistent with PTEN loss of function being a common feature of TNBC and its cell models and provides validation of our approach.

Targeting mitosis and cell division as an anti-cancer strategy is not novel and has been the basis of successful systemic microtubule targeting chemotherapies such as the taxanes, vinca alkaloids, and eribulin. However, the impact on patient benefit has been constrained by the fact these agents have substantial normal tissue toxicity due to effects in non-malignant tissues with high cellular turnover leading to a narrow therapeutic window. In contrast the specificity of the dependency on the KIFC1 kinesin to centrosome-amplified cancer cells offers the possibility of a potentially druggable malignant cell-specific target and a mechanism-based patient selection biomarker of centrosome abnormality that can be applied to routinely collected formalin-fixed tumors. In a non-malignant setting, KIFC1 is not required for the faithful division of somatic cells^44^ suggesting its inhibition would be well tolerated by non-malignant tissues in patients; however, as it is known to play a role in spermiogenesis and oocyte development^45^ possible germ cell toxicity may be anticipated. In a malignant setting, *KIFC1* expression has been correlated with poor prognosis in breast cancer^46^, and higher level expression is observed in ovarian adenocarcinoma patients^47^ and in other cancer types, including glioblastoma, lung, colon, and cervical tumor samples when compared to corresponding normal tissues. Although the centrosome clustering-independent mechanisms have been suggested to be relevant to KIFC1 addiction in breast cancer^48^, we find across multiple model systems that there is a strong dependency on the centrosome clustering function of KIFC1 by detection of mitotic catastrophe and correlation of centrosome amplification with sensitivity to KIFC1 depletion. KIFC1 expression in non-small cell lung carcinoma was found to be highly predictive of the development of brain metastasis in both early and advanced disease^49^ suggesting association of expression with highly aggressive forms of common cancers.

Effects of KIFC1 on treatment resistance may contribute to poor prognosis. It has been shown that *KIFC1* overexpression is correlated with resistance to the mitotic spindle-stabilizing agent docetaxel^50^. A possible explanation of these findings is based on recent evidence showing that the mechanism of action of clinically relevant doses of paclitaxel is through induction of multipolar mitosis^51,52^ raising the possibility that a KIFC1-dependent mechanism of microtubule-organizing center re-clustering may be involved in the development of taxane resistance.

Our data show that inhibiting KIFC1 in BCCL models leads to cell population growth arrest both in in vitro culture and in vivo models, and that this is specific to centrosome-amplified cells. This is supported by previous findings in non-breast cancer isogenic cell line model systems with artificially induced centrosome amplification^36^. Centrosome amplification is a well-characterized phenomenon that is specific to cancer cells, first described by Theodor Boveri over a 100 years ago^53^. Since then studies have identified that centrosome amplification is sufficient to initiate tumorigenesis^34,54^ and that centrosome amplification can also mimic the effects of oncogenes in triggering cellular invasion^55^. Therefore, targeting centrosome-amplified cells by KIFC1 inhibition would be expected to have effects in many cancer types. To identify tumors with centrosome amplification we have also presented the preliminary development of a method, PCAB, which quantifies centrosome features in excess of those of normal tissue cells that might be used to stratify patients and predict a population that appear to have adverse prognosis and may benefit from inhibition of the KIFC1 kinesin motor. We show that this PCAB score developed in malignant and normal breast tissue identifies a large proportion of TNBC patients with centrosome amplification who have poor prognosis and associates with KIFC1 addiction in breast cancer cell models.

Our in vivo studies showed a significant growth inhibition of KIFC1-depleted centrosome-amplified cell xenografts (two-way ANOVA with Sidak’s multiple comparisons test). The fact that our inducible shRNA in vivo model depletes but does not completely ablate the expression of KIFC1 may explain why inducible expression of KIFC1 shRNA impairs but does not eradicate the tumor. This highlights the potential value in now developing potent and selective small molecule KIFC1 motor inhibitor compounds and examining their efficacy in preclinical models. We have demonstrated a synergistic effect between KIFC1 silencing and cisplatin treatment, a therapy that induces centrosome amplification in cancer cells, on long-term clonal growth in culture and in vivo xenografts growth suggesting potential for combination approaches with a standard of care chemotherapy increasingly used in TNBC.

In summary, our work describes an integrated CNA and gene expression-driven gene dependency identification and functional validation approach that identifies novel malignant cell selective addictions, potential targetable genes or pathways and associated patient selection biomarkers in TNBC. We reveal a number of genes, biological processes, and clusters of interacting proteins that merit further investigation. In particular, we identify and mechanistically validate KIFC1, a potentially druggable kinesin, as a highly selective malignant cell target, with mechanistic evidence of synergy in combination with cisplatin. Furthermore, we developed a potential patient selection centrosome abnormality biomarker appropriate for analysis of formalin-fixed tumor material that is associated with KIFC1 addiction.

## Methods

### Patient demographics

All procedures performed in studies involving human participants were in accordance with the ethical standards of the institutional and/or national research committee and with the 1964 Helsinki declaration and its later amendments or comparable ethical standards. Informed consent was obtained from all human participants. Access to pseudo-anonymized samples and clinical data was obtained in accordance with the principles of the Guy’s and St Thomas’ Research Tissue and Data Bank (REC No 07/H0804/131). Fresh-frozen needle macro-dissected invasive ductal carcinomas obtained from 191 breast cancer patients with no prior therapy at diagnosis and control tissue from reduction mammoplasties, and peripheral blood lymphocytes were obtained from King’s Health Partners Cancer Biobank. The patient demographics and clinico-pathological information have been previously published^14,15,56^ and are described in Supplementary Data 1.

### Immunohistochemistry on tumor samples

IHC expression of ER, PR, HER2, EGFR, and CK5/6 were assessed on triplicate tissue microarrays (TMA) and reviewed by two pathologists. The tissue was formalin-fixed, processed and paraffin-embedded using routine protocols. Three-micron sections were cut and stained with hematoxylin and eosin (Dako). Standard IHC protocols were used. Antigen retrieval was carried out using citric acid buffer pH 6 (Dako). For visualization of nuclei, hematoxylin counterstain was used. Secondary antibodies conjugated to horseradish peroxidase (HRP) (Dako) were used for visualization with 3,3-diaminobenzidine (DAB) (Dako), according to manufacturer’s protocol.

### Microarray-based gene expression and copy number profiling

Gene expression and copy number profiles were generated using Affymetrix Human Exon 1.0ST and SNP6.0 arrays (E-MTAB-5270 and E-MTAB-2626) and data was processed using specific gene expression and copy number pipelines from Aroma Framework^15,16,39,56–59^. The data comprised 140 ER-negative and HER2-negative, 21 HER2-positive, 21 ER-positive breast carcinomas, and 9 RNA samples derived from organoids of reduction mammoplasties. ER and HER2 IHC-based expression levels were confirmed by gene expression for each sample.

### Target ID data platform

A gene-centric database was compiled (Supplementary Data 2), encompassing information from five different categories: (A) gene expression block, (B) copy number block, (C) copy number-gene expression association block, (D) clinical annotation block, and (E) gene annotation block using publicly available databases such as COSMIC^17^, the Membranome^18^, the Druggable Genome^19^, Secretome^20^, CAN genes^21^, and Kinome^22^. At our first filter, we eliminated genes with weak correlation between gene expression and copy number. We therefore identified genes which had a gain (absolute copy numbers ≥ 2.38 based on cbs-segmented copy number) in at least 10% of TNBC cases, and which gene expression showed a Spearman’s rank correlation of at least 0.3 with a *p*-value of at least 0.01.

These genes were included in our Target ID platform, consisting of (A) the “gene expression block”, capturing the fold change and the significance of differential gene expression between TNBC and normal mammary gland tissue, all tumors and normal mammary gland tissue as determined by the *limma* analyses^60^, and the frequency of samples in which the respective gene had expression levels twice as high as in the normal mammary gland tissue. (B) The “copy number block”, reporting for each gene the frequency of copy number gain/amplification in TNBCs, the average copy number levels in TNBC, and the focality of CNA determined by GISTIC^61^. (C) The “copy number-gene expression association block”, states the Spearman’s rank correlation between each genes copy number and gene expression; and a multi-Mann–Whitney *U* test using categorical copy number states as the grouping variable and the expression of the gene as the dependent variable as previously described^62^. Benjamini and Hochberg adjusted *p* < 0.05 were considered significant^63^. (D) The “clinical annotation block”, reporting fold change and *p*-value of differential gene expression between TNBC with and without recurrences as determined by *limma*^60^. (E) The “ gene annotation block” collated publicly available information such as: gene description; mutational status in cancer derived from COSMIC^17^; cell surface protein location and length of the extracellular domain retrieved from Membranome^18^; druggability potential retrieved from “the druggable genome”^19^; other information such as Secretome^20^, CAN genes^64^, and Kinome^22^. Supplementary Data 2 lists all of these features, their block affiliation, block description, priority weighting, thresholds, and scores.

The feature weighting module assigned scores for each feature in the binning approach. If the feature value is smaller than threshold1, then it is assigned a 0 score, if it lies between theshold1 and threshold2 it is assigned Score 1, if it is greater than threshold2 it gets Score 2. The aggregated scores are reported for each individual gene. The features are then combined to make five different blocks of features and a limit on total block score is assigned for each block in order to minimize the bias towards any specific block. The aggregated scores from each block are then normalized with respect to the block limit and their summation is reported as block normalized score.

### Cell lines

BCCLs were obtained from the ATCC and HMEC from Life Technologies. Growth conditions were as recommended by the suppliers. Cells were authenticated by short tandem repeat (STR) analysis and matched to the German Collection of Microorganisms and Cell Cultures (DSMZ), and they were used for no more than 25 passages after STR typing. Mycoplasma tests were routinely performed using MycoAlert Mycoplasma Detection Kit (Lonza). Although MCF7 and BT20 are included in the database of commonly misidentified cell lines, they were authenticated by STR, and we included them in our work as part of a comprehensive validation.

### Breast cancer cell line gene expression data

Gene expression for 25 BCCLs based on the Illumina Human WG-6v2.0 and for 27 BCCLs based on the Affymetrix Human Exon 1.0ST microarrays were reported previously^24,39^, with 20 BCCLs common to both data sets. Both normalized gene expression cohorts were independently median-centered.

### Target ID data platform and algorithm

A gene-centric database was compiled (Supplementary Data 2). This encompassed information from five different categories: (A) gene expression block, (B) copy number block, (C) copy number-gene expression association block, (D) clinical annotation block, and (E) gene annotation block using publicly available databases such as COSMIC^17^, the Membranome^18^, the Druggable Genome^19^, Secretome^20^, CAN genes^21^, and Kinome^22^.

To identify potential CNA-regulated addiction genes for TNBC malignant phenotypes, genes with cbs-smoothed Log_2_ ratio ≥2.38 in ≥10% TNBCs were selected. We then selected those with Spearman’s rank correlation between expression and copy number data ≥0.30 and *p* > 0.01 in the TNBCs in our discovery cohort were taken forward. Next genes were assigned a score that was derived through a custom defined weighted evaluation of the five blocks in the Target ID Data Platform (Supplementary Data 3). All code for the algorithm used was implemented using the R statistical language.

### RNAi-based functional validation

Gene “lots” and their assigned cell lines were established as follows: the three highest and lowest expressing BCCLs for each gene were determined. Those genes having at least one cell line in common, among the highest and lowest expressing cell lines, were grouped into gene “lots”. Thus, the candidate genes were divided into five lots each to be tested in 6 to 9 breast cell lines, resulting in a total of 16 BCCLs, and on one non-malignant cell line HMEC (Supplementary Figure 1).

The “top 10” gene set was grouped into a separate lot and siRNA-mediated knockdown was carried out by transfection of three siRNAs (Ambion) independently at a concentration of 50 nM. Cells were plated in 96-well plates and cell viability assays were performed in eight cell lines over three rounds using CellTiter-Blue Cell Viability Assay (Promega) over 6-days and gene knockdown by each siRNA was assessed by RT-PCR.

For the remaining five lots, siRNA-mediated knockdown was carried out by transfection of pools of four siGenome siRNAs (Dharmacon) targeting the same gene, at the total concentration of 50 nM. Cell viability assays were performed in three rounds for each gene lot using the CellTiter-Blue Cell Viability Assay (Promega) over 6-days. After normalization against the plate median and correlation between the three rounds analyzed (Supplementary Data 10), the data from the three rounds were pooled and the mean was plotted ± SEM. siRNA against PLK1 and scrambled “non-silencing” siRNAs were used as positive and negative controls, respectively.

The effect on cell viability was expressed as NPI where:

$${\mathrm{NPIx}}\% = \left( {x-{\mathrm{\mu }}_{{\mathrm{neg}}}} \right)/\left( {\mu _{{\mathrm{pos}}}-{\mathrm{\mu }}_{{\mathrm{neg}}}} \right)\times 100$$

NPIx is the NPI of the *x* sample and *µ*_pos_ and *µ*_neg_ are the averages of positive or negative controls, respectively, within that plate.

From the top ten gene list, genes having at least two siRNAs with KD ≥ 70% showing an NPI < 18.01% in HMEC and an NPI ≥ 18.01% in at least two malignant cell lines were considered as validated. For the remaining lots, genes showing NPI < 18.01% in HMEC and an NPI ≥ 18.01% in at least two malignant cell lines, or if they had an NPI ≥ 18.01% in HMEC then have an NPI < 18.01% in at least two malignant cell lines and an NPI ≥ 18.01% in at least two malignant cell lines were considered as passing the primary functional validation experiment.

### Quality control of the siRNA candidate screen

Prior to carrying out the functional validation experiments, siRNA transfection conditions were optimized for each cell lines such that PLK1 siRNAs produced a >70% reduction in CellTiter-Blue readings after 6 days compared to non-silencing siRNA with minimal transfection reagent toxicity. Experiments were carried out in 96-well plates, excluding the use of more external rows and columns, to avoid edge effects (Supplementary Figure 2b). To achieve higher data robustness, multiple positive and negative controls were added to each plate and each round of the validation consisted of triplicate plates. A good separation between positive and negative controls was also seen across all cell lines (Supplementary Figure 2b). Position dependent effects were ruled out. Valid experiments had a *Z’* factor ≥0.3 and replicates were excluded if the *r*^2^ between them and other replicates were <0.5. Overall the mean *r*^2^ values for all replicates within the analysis were 0.7, indicative of good quality data (Supplementary Data 4).

$$Z^{\prime} {\hbox{-}} {\mathrm{ factor}} = 1 - \frac{{3{\mathrm{SD}}\,{\mathrm{of}}\,{\mathrm{pos}}\,{\mathrm{controls}} + 3{\mathrm{SD}}\,{\mathrm{of}}\,{\mathrm{neg}}\,{\mathrm{controls}}}}{{{\mathrm{mean}}\,{\mathrm{of}}\,{\mathrm{pos}}\,{\mathrm{controls}} - {\mathrm{mean}}\,{\mathrm{of}}\,{\mathrm{neg}}\,{\mathrm{controls}}}}$$

### Secondary functional validation

Single oligo deconvolution of the pooled siRNA from the primary functional validation experiments were performed by grouping the genes in “lots”, each to be tested against a small number of cell lines which appeared in the primary validation to be relatively more addicted to some of the genes in that lot. Each of the four individual oligos were assessed for both knockdown efficiency, by RT-PCR, and effect on cell viability and the results integrated. Genes having more than one individual siRNA sequence showing NPI < 18.01% despite KD ≥ 70% were considered as a fail. Those genes for which at least two siRNAs showed KD ≥ 40% and an NPI ≥ 18.01% were considered as validated.

### Centrosome scoring

Confocal imaging of a panel of BCCLs generated centrosome amplification score. Cells were double immunostained with α-IAK1 (BD Biosciences) and α-CP110 (gift from E. Nigg) for centrosomes and centrioles, respectively. Cells were counted as centrosome-amplified if they had more than two centriole markers per centrosome and/or if they had more than two centrosomes per cell. Centrosome amplification score was calculated as the percentage of centrosome-amplified cells. For cisplatin-treated cells, centrosomes were scored using the Perkin Elmer Operetta High Content Imaging System (Perkin Elmer) and Harmony (Perkin Elmer).

### Multipolar mitosis assay

Cells were plated in 96-well plates and transfected with individual oligonucleotides (10 nM) including non-silencing negative control. Knockdown of target gene was confirmed by western blot 72 h post-transfection and cells were fixed in methanol and stained with α-IAK1 as a mitotic spindle marker. Image acquisition was performed using the Perkin Elmer Operetta High Content Imaging System (Perkin Elmer) and analyzed using Harmony (Perkin Elmer). Mitotic cells with more than two mitotic spindle poles were scored as multipolar and the percentage of multipolar mitoses was calculated from all visible mitoses in a well of a 96-well plate (*n* > 100 for each replicate).

### Time-lapse microscopy

MDA-MB-231 stable inducible NT and KIFC1 shRNA cells were transduced a constitutive lentiviral vector expressing a mCherry-tagged histone H2B as a fluorescent DNA marker. The cells were treated with doxycycline for a 72-h period to ensure full expression of the shRNA before the start of the live-cell imaging. Image acquisition was performed using Nikon Eclipse TE2000 with a Hamamatsu Digital Camera. Images were acquired with a ×20 objective and images were taken every 4 min for 50 h. Mitotic cells were scored as either bipolar or multipolar and normal or apoptotic.

### Animal studies

All applicable international, national, and/or institutional guidelines for the care and use of animals were followed. All procedures performed in studies involving animals were in accordance with the ethical standards of the institution or practice at which the studies were conducted. All animal experiments were approved by the King’s College London Institutional Committees on Animal Welfare (Animal Welfare and Ethical Review Body) and in compliance with the United Kingdom Home Office Animals Scientific Procedures Act, 1986. Female CD-1 Nu/Nu mice were obtained from Charles River UK Ltd. Procedures were carried out after 20–35 days of age, mice were maintained behind a barrier facility and handled in accordance with local guidelines. One million MDA-MB-231 or HCC1954 stable inducible NT and KIFC1 shRNA cells were injected into the right inguinal mammary fat pad of mice following standard procedures. When tumors reached 2 × 2 mm (4.2 mm^3^) (as assessed by palpation and caliper measurement) mice were randomized into two groups and one group were fed chow ad libitum containing Doxycycline at 625 mg kg^−1^ (Harlan Teklad Diets). Tumor growth was monitored over time (assessed by palpation and caliper measurement). When the first control tumors reached 10 mm in diameter, all mice for that experiment were culled and tumors were excised and snap-frozen or processed for IHC (FFPE). Tumor volume was calculated using the formula: *V* = (*π* × length × width^2^/6), where the length is the largest tumor diameter and width is the perpendicular diameter. Statistical analysis was performed using Prism.

### Immunohistochemical analysis

All primary antibodies used in this study are shown in Supplementary Data 12. All the histological samples were scanned at ×20 (0.46 µm per pixel), except with Pericentrin staining which were scanned at ×40 (0.25 µm per pixel) digital magnification using Hamamatsu Nanozoomer 2.0 HT (Hamamatsu). The IHC assessment was performed using semi-automated Image Analysis software HistoQuest 4.0 (TissueGnostic).

Pericentrin staining was assessed in mitotic cells by a trained histopathologist. The mitoses were scored as having either normal centrosomes or abnormal centrosomes. Abnormal centrosomes were defined by either size (twice the diameter of centrosomes in normal breast epithelium) or number (>2) (Supplementary Figure 5c). Mitoses were further classified by polarity based on the orientation of DNA and centrosomes in a cell. Mitotic cells with abnormal centrosomes fell within the category of bipolar, multipolar, or those with diffuse pericentrin and no polarity.

### Pericentrin abnormality score

HistoQuest image analysis software was set up to count the number of pericentrin events and staining area per event for cell line pellets, whole tissue sections and TMAs. PCAB score was developed using by using a cut-off for normal centrosome size (7 µm^2^) defined by analysis of pericentrin centrosome size in normal breast tissue. The score is the number of abnormal pericentrin bodies as a percentage of total bodies within a selected field. For whole sections the tumor region was selected as the field of interest. For cell pellets and TMAs the entire section or core, respectively, was selected as the field of interest with at least 20 pericentrin bodies being score per case (average 440 pericentrin bodies being scored per case).

### KIFC1 si and shRNA sequences

KIFC1 siRNA #1: GGACUUAAAGGGUCAGUUA, KIFC1 siRNA #2: GUGCUAAGAUGCUCAUGUU, KIFC1 siRNA #3: GGAGCUCACUGUCACCAAU, KIFC1 siRNA #4: UGACCUAAAUGCAGAACUA, KIFC1 siRNA #5: CUCUACGCUUUGCCUCCAA, KIFC1 siRNA #6: GUAGAGAUCUACAAUGAGA, KIFC1 shRNA: AAGCTACGTAGAGATCTACAAT.

### Generation of stable inducible NT and KIFC1 shRNA cell lines

Oligonucleotides with both NT and KIFC1-targeting (KIFC1 shRNA) shRNAs with flanking AgeI and EcoRI restriction sites were cloned into the Tet-pLK0-puro plasmid (a gift from Dmitri Wiederschain via Addgene). Lentiviral particles were subsequently produced by transfecting HEK293T cells with the plasmid and lentiviral packaging vectors pSPAX2 and pMD2.G. Next, CAMA1, BT20, HCC1954, and MDA-MB-231 were infected with the lentivirus and then cultured in the presence of 1.5 μg ml^−1^ puromycin. Knockdown was confirmed in vitro by addition of 1 μg ml^−1^ doxycycline for 72 h followed by Western blot.

### Colony formation assays

Surviving fraction of cells was analyzed using colony formation assays. Cell lines expressing the inducible vectors were generated as above. Cells were plated at low density and shRNA expression was induced by addition of doxycycline. Knockdown of target gene was confirmed by Western blot 96 h post-transduction and colonies were fixed with ice-cold methanol and stained with 0.5% crystal violet at 14 days post-transduction. Colony size was defined as a minimum of 50 cells and colonies were counted using BIO-RAD XRS+system (BIO-RAD) and Image Lab XRS+software (BIO-RAD).

### Statistical analysis

Gene expression and copy number statistical analyses were performed in the R environment as described above. For in vitro studies, no samples were processed and then excluded; all completed experiments are reported. Unpaired two-sided *t*-tests and one-way ANOVA with Tukey’s multiple comparison were performed using GraphPad Prism software for analysis of all in vitro and in vivo studies. For in vivo studies, we estimated that we would need at least six samples per treatment group to see an effect, for a power of 80% and for a probability of Type I error (*α*) = 0.05. Experiments were repeated at least twice to confirm treatment response. The total number of mice per group is indicated. Mice were excluded from the study if body weight was reduced during treatment by more than 15% as compared to that at the start of treatment. Investigators were blinded to the group allocation during the experiment and drug treatment. Investigators were also blinded when assessing the outcome by IHC. Mice were randomized to treatment groups when tumors reached a predetermined diameter on a per experiment basis, as described above. The sample size for all in vitro experiments were not chosen with consideration of the power needed to detect a pre-specified effect size. For each data set, the data meet the assumptions of the statistical test used, as determined by distribution and variance.

### Data availability

All data is available from ArrayExpress (https://www.ebi.ac.uk/arrayexpress/) under following accession codes (E-MTAB-5270 and E-MTAB-2626), and can be interrogated via our web portal upon request.

## Electronic supplementary material

Supplementary Information

Description of Additional Supplementary Files

Supplementary Data 1

Supplementary Data 2

Supplementary Data 3

Supplementary Data 4

Supplementary Data 5

Supplementary Data 6

Supplementary Data 7

Supplementary Data 8

Supplementary Data 9

Supplementary Data 10

Supplementary Data 11

Supplementary Data 12

Supplementary Movie 1

Supplementary Movie 2

Supplementary Movie 3
