# Supplementary material for: Integrated genomics and functional validation identifies malignant cell specific dependencies in triple negative breast cancer
Source: Nat Commun. 2018 Mar 13;9:1044. doi: 10.1038/s41467-018-03283-z (PMC5849766; doi:10.1038/s41467-018-03283-z)
Supplement: Supplementary file 1 — Supplementary Information [file 41467_2018_3283_MOESM1_ESM.pdf]

## Supplementary Figures

**a**

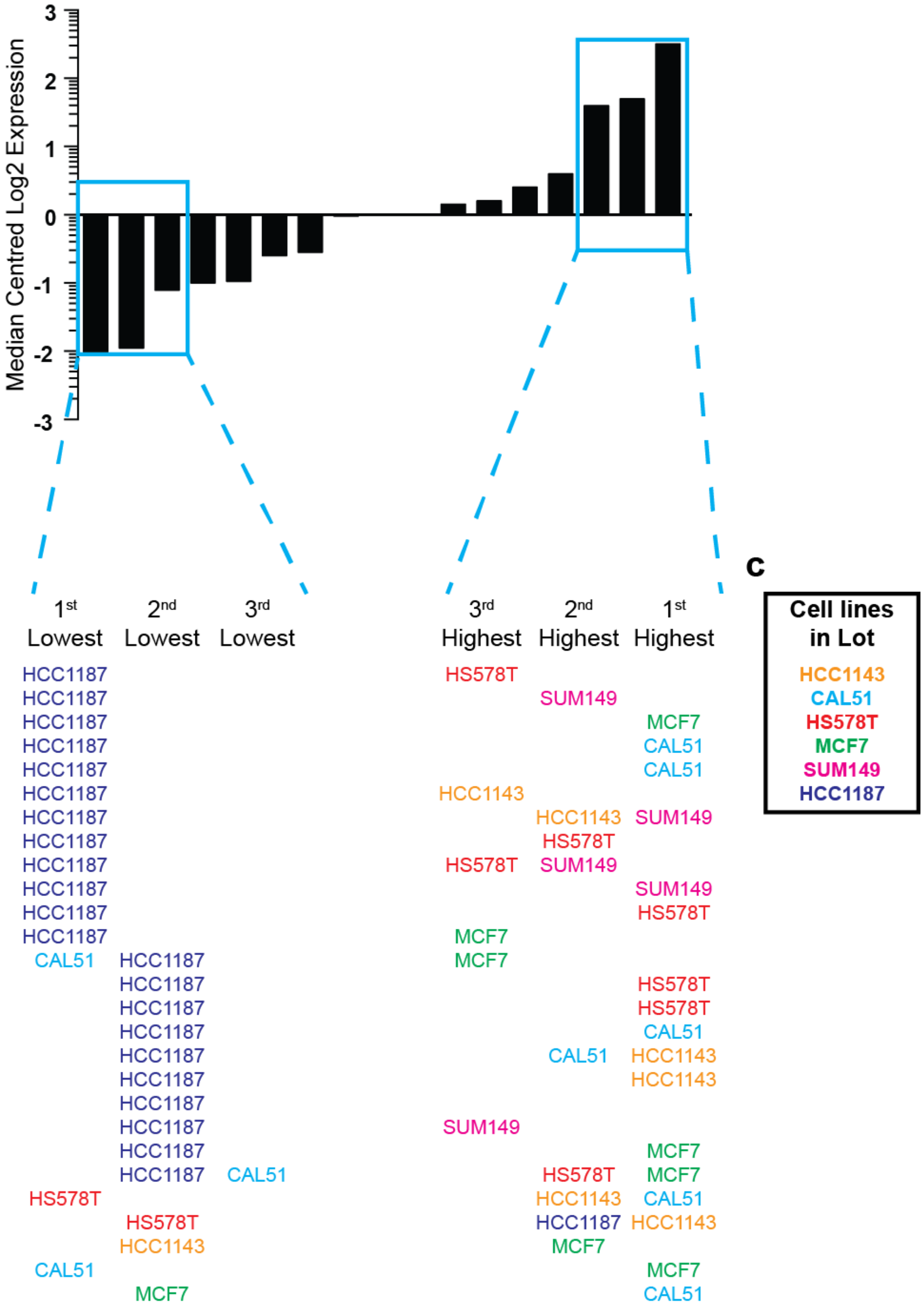


**b**

| Genes in Lot | 1st Lowest | 2nd Lowest | 3rd Lowest | 3rd Highest | 2nd Highest | 1st Highest |
|---|---|---|---|---|---|---|
| NUF2 | HCC1187 | | | HS578T | | |
| ZNF260 | HCC1187 | | | | SUM149 | |
| CENPF | HCC1187 | | | | | MCF7 |
| ELF5 | HCC1187 | | | | | CAL51 |
| SCNM1 | HCC1187 | | | | | CAL51 |
| APH1A | HCC1187 | | | HCC1143 | | |
| VPS45 | HCC1187 | | | | HCC1143 | SUM149 |
| GPR89B | HCC1187 | | | | HS578T | |
| GPR89C | HCC1187 | | | HS578T | SUM149 | |
| CTSK | HCC1187 | | | | | SUM149 |
| EZH2 | HCC1187 | | | | | HS578T |
| STMN1 | HCC1187 | | | MCF7 | | |
| RRM1 | CAL51 | HCC1187 | | MCF7 | | |
| LANL2 | | HCC1187 | | | | HS578T |
| LIN9 | | HCC1187 | | | | HS578T |
| OAS1 | | HCC1187 | | | | CAL51 |
| S100A7 | | HCC1187 | | | CAL51 | HCC1143 |
| PLCH1 | | HCC1187 | | | | HCC1143 |
| DHTKD1 | | HCC1187 | | | | |
| PSMD4 | | HCC1187 | | SUM149 | | |
| CTSS | | HCC1187 | | | | MCF7 |
| GBAS | | HCC1187 | CAL51 | | HS578T | MCF7 |
| SULF1 | HS578T | | | | HCC1143 | CAL51 |
| PTPRC | | HS578T | | | HCC1187 | HCC1143 |
| PRKDC | | HCC1143 | | | MCF7 | |
| BUB1B | CAL51 | | | | | MCF7 |
| NPAL2 | | MCF7 | | | | CAL51 |

**c**

**Cell lines in Lot**

**HCC1143**
**CAL51**
**HS578T**
**MCF7**
**SUM149**
**HCC1187**

Supplementary Fig. 1 Patel *et al.*,

**Supplementary Figure 1 Gene and cell line assignment into lots.**

**a.** Example of median centered Log2 expression of gene across panel of 17 breast cell lines (described in Materials and Methods). Highlighted are 3 cell lines with the lowest and 3 cell lines with the highest relative expression. **b.** Example gene lot showing the occurrence of each cell line within the 3 lowest and highest relative expression groups. **c.** Example of cell lines used in this particular lot. The minimum number of cell lines which appeared at least once among the 3 highest and 3 lowest, for each gene in any of the lots, was used for the experimental validation of that specific gene lot.

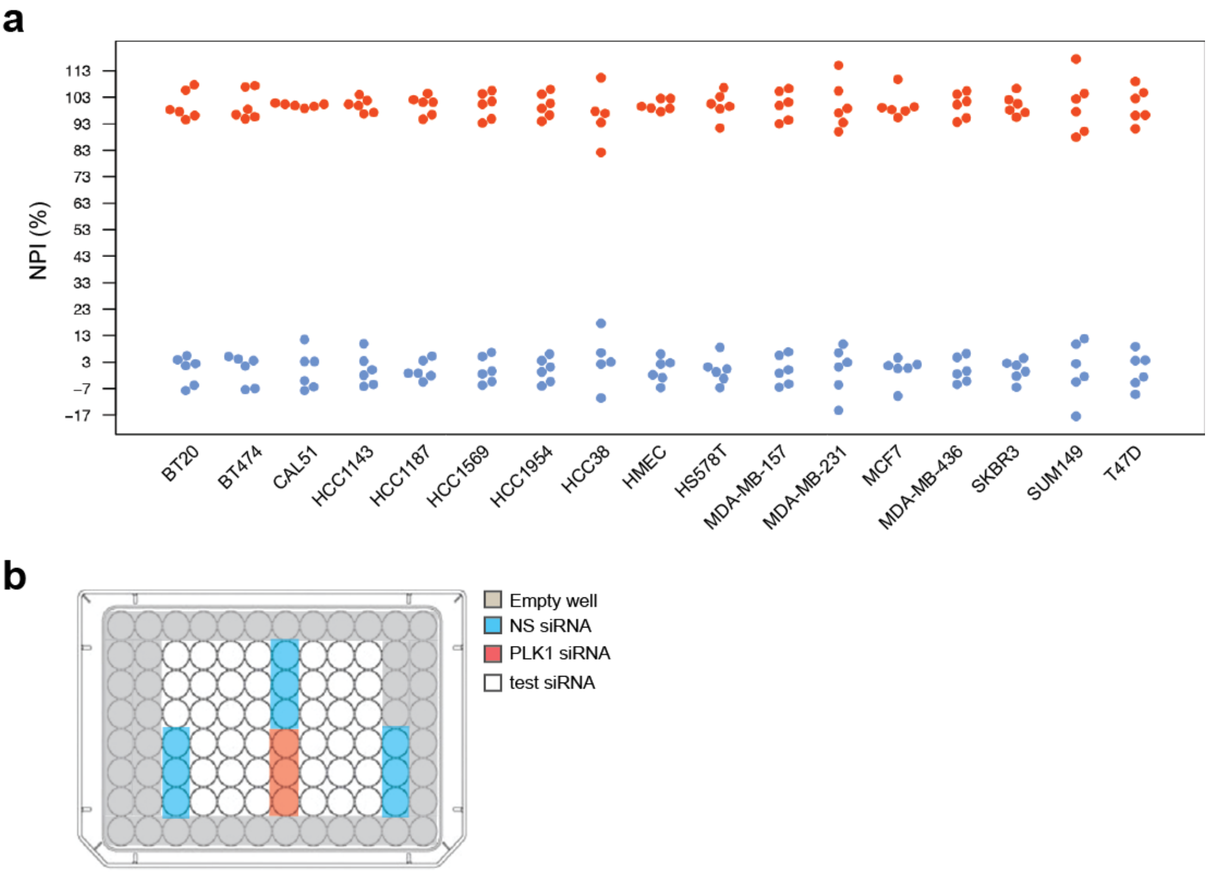


Supplementary Fig. 2 Patel *et al.*,

**Supplementary Figure 2 Quality control of functional RNAi validation experiment.**

**a.** Distribution of a subset of NPI values of positive and negative controls across all cell lines used in functional RNAi experiment. **b.** Plate template showing positioning of controls and samples.

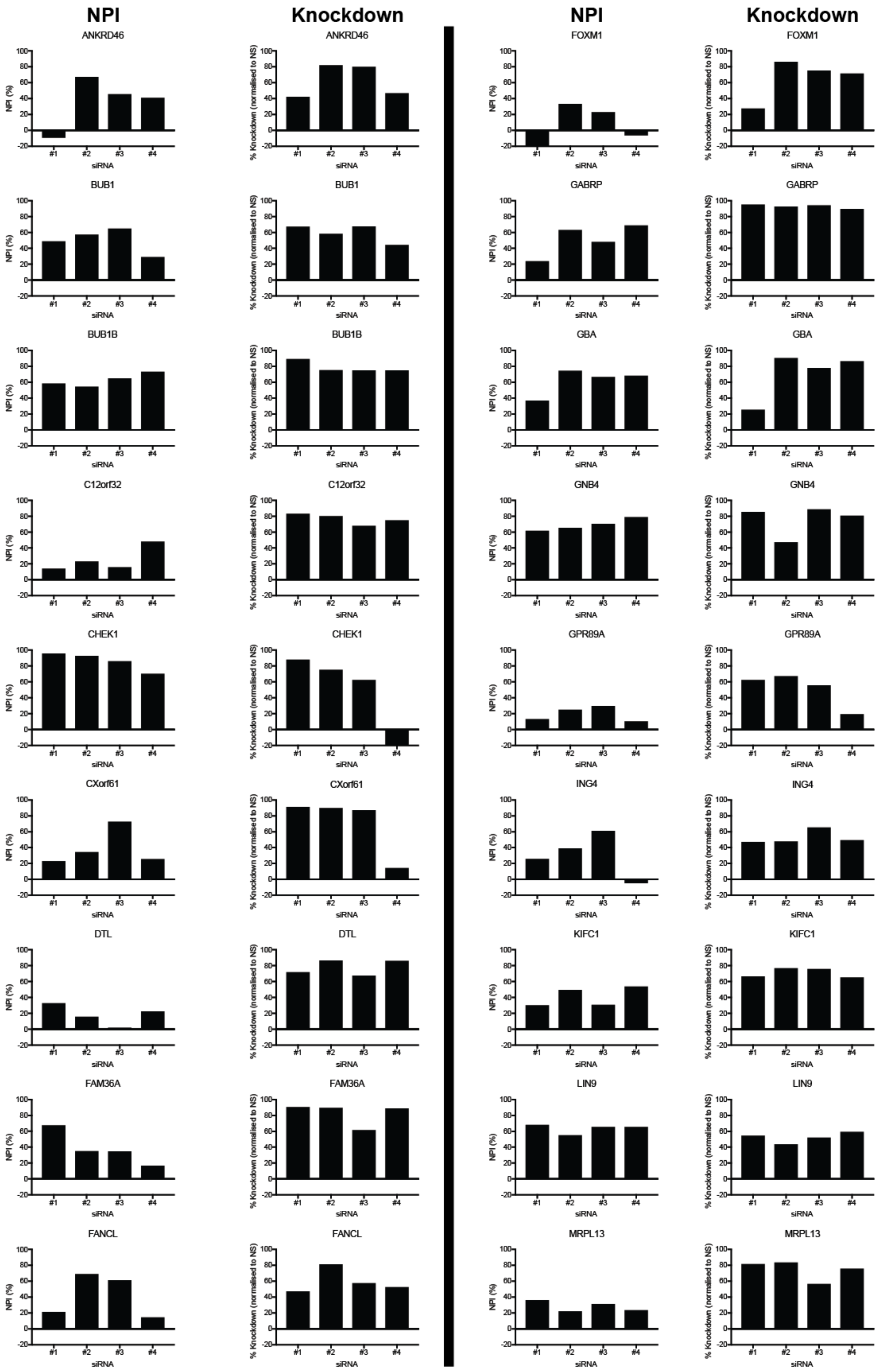
NPI
Knockdown
NPI
Knockdown
ANKRD46
FOXM1
BUB1
GABRP
BUB1B
GBA
C12orf32
GNB4
CHEK1
GPR89A
CXorf61
ING4
DTL
KIFC1
FAM36A
LIN9
FANCL
MRPL13
NPI (%)
% Knockdown (normalised to NS)
siRNA
#1
#2
#3
#4

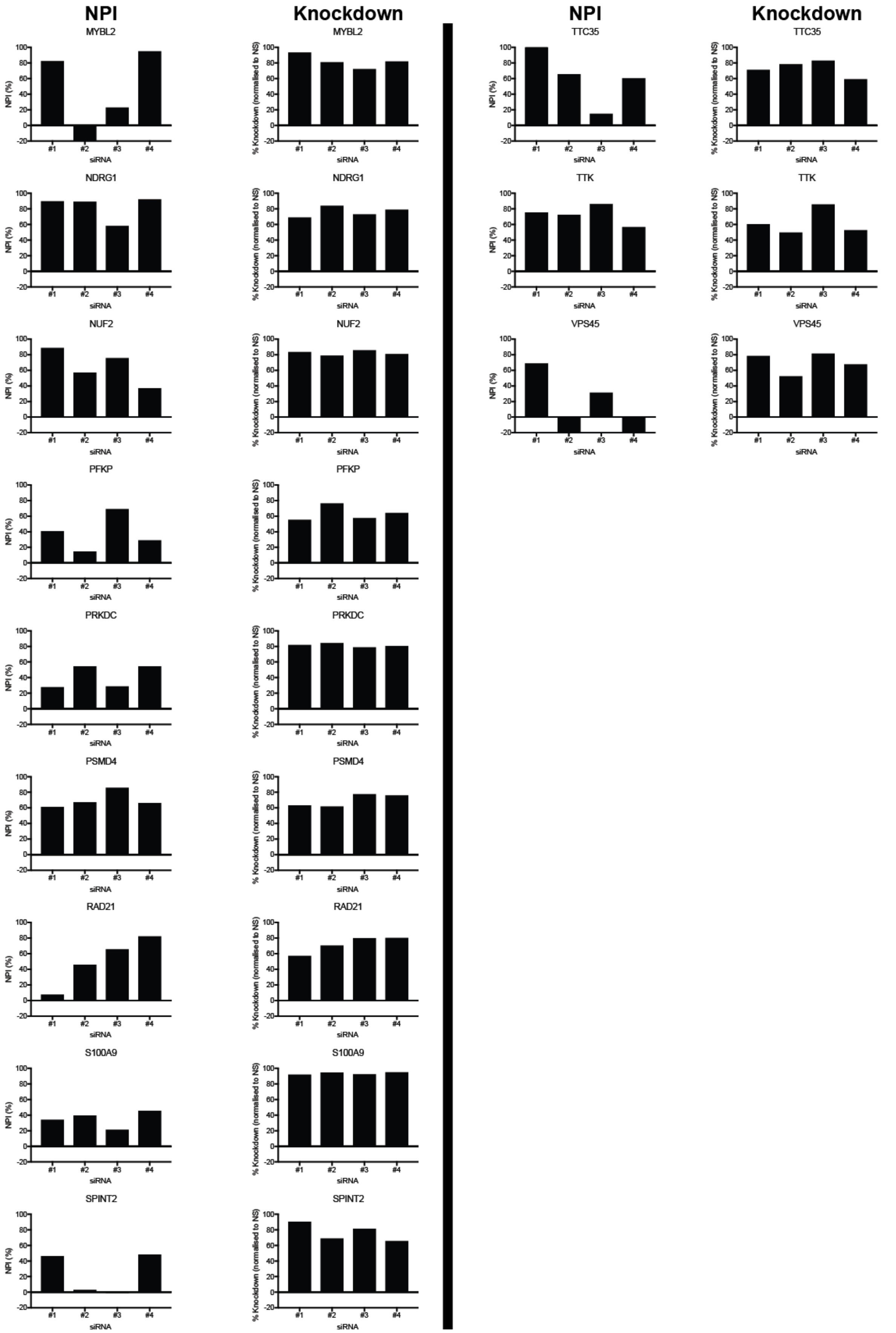
NPI
Knockdown
NPI
Knockdown
MYBL2
TTC35
NDRG1
TTK
NUF2
VPS45
PFKP
PRKDC
PSMD4
RAD21
S100A9
SPINT2
NPI (%)
% Knockdown (normalised to NS)
siRNA
#1
#2
#3
#4

**Supplementary Figure 3 Normalized percent inhibition and mean percentage knockdown for further validation of hits.**

Left, Normalized percent inhibition using four independent siRNA species against gene of interest. Right, mean percentage knockdown of gene of interest by qPCR compared to non-silencing control for four independent siRNAs. Cell lines for each gene are shown in Supplementary Data 9.

| | | | | | | | | | | | | | | | | |
|---|---|---|---|---|---|---|---|---|---|---|---|---|---|---|---|---|
| ANKRD46 | | | | | | | | BT20 | HMEC | HCC38 | SUM149 | T47D | HCC1954 | CAL51 | | |
| BUB1 | | | | | | | | SKBR3 | HMEC | T47D | BT20 | HCC1143 | CAL51 | MDAMB231 | | |
| BUB1B | | | | | | | | MCF7 | HMEC | MDAMB436 | HCC1187 | SUM149 | HCC1143 | CAL51 | | |
| C12orf32/RHNO1 | | | | | | | | | HMEC | HCC38 | SKBR3 | MDAMB231 | BT474 | CAL51 | MDAMB436 | |
| CHEK1 | | | | | | HCC38 | BT20 | HCC1954 | HMEC | CAL51 | T47D | SUM149 | | | | |
| CXorf61 | | | | | | | | CAL51 | HMEC | T47D | SKBR3 | BT20 | MDAMB231 | HCC1143 | | |
| DTL | | | | | | | | | HMEC | BT20 | MDAMB231 | SKBR3 | CAL51 | T47D | HCC1143 | |
| FAM36A | | | | | | | HCC1954 | BT20 | HMEC | SUM149 | T47D | CAL51 | HCC38 | | | |
| FANCL | | | | | | | T47D | MDAMB231 | HMEC | BT20 | SKBR3 | CAL51 | HCC1143 | | | |
| FOXM1 | | | | | | | | | HMEC | MCF7 | HCC1143 | CAL51 | HCC1187 | MDAMB436 | SUM149 | |
| FZD6 | | T47D | SKBR3 | MDAMB157 | HCC1569 | BT20 | HCC1143 | MCF7 | HMEC | | | | | | | |
| GABRP | | | | | CAL51 | T47D | MDAMB231 | SKBR3 | HMEC | BT20 | HCC1143 | | | | | |
| GBA | | | | SUM149 | MDAMB157 | CAL51 | HCC1143 | HS578T | HMEC | HCC38 | MCF7 | HCC1569 | MDAMB436 | | | |
| GNB4 | | | | | BT20 | CAL51 | SKBR3 | MDAMB231 | HMEC | HCC1143 | T47D | | | | | |
| GPR89A | | | | | | SUM149 | HCC1143 | CAL51 | HMEC | MCF7 | MDAMB436 | HCC1187 | | | | |
| ING4 | | | | | | | | | HMEC | HCC1954 | T47D | HCC38 | CAL51 | BT20 | SUM149 | |
| KIFC1 | | | | | | | | | HMEC | SKBR3 | T47D | BT20 | CAL51 | MDAMB231 | HCC1143 | |
| LIN9 | | | | | | | | | HMEC | MCF7 | HCC1143 | CAL51 | MDAMB436 | HCC1187 | SUM149 | |
| MASTL | | | | | | | MDAMB157 | MCF7 | HMEC | SKBR3 | T47D | HCC1569 | BT20 | HCC1143 | | |
| MRPL13 | | | | | | | BT474 | HCC38 | HMEC | MDAMB231 | CAL51 | MDAMB436 | SKBR3 | | | |
| MYBL2 | | | | | | | | | HMEC | BT474 | MDAMB231 | MDAMB436 | SKBR3 | CAL51 | HCC38 | |
| NCSTN | | | | MDAMB157 | HCC1143 | MCF7 | BT20 | HCC1569 | HMEC | SKBR3 | T47D | | | | | |
| NDRG1 | HCC38 | MDAMB157 | CAL51 | MCF7 | MDAMB436 | HCC1143 | SUM149 | HS578T | HMEC | HCC1569 | | | | | | |
| NUF2 | | | | | | | | | HMEC | MCF7 | HCC1143 | SUM149 | CAL51 | HCC1187 | | |
| PFKP | | | | BT20 | CAL51 | HCC38 | T47D | HCC1954 | HMEC | SUM149 | | | | | | |
| PRKDC | | | | | | | | MCF7 | HMEC | MDAMB436 | SUM149 | CAL51 | HCC1187 | HCC1143 | | |
| PSMD4 | | | | | | | | | HMEC | SUM149 | CAL51 | MCF7 | HCC1143 | HCC1187 | MDAMB436 | |
| PTK2 | | | | | | | | MDAMB157 | HMEC | MCF7 | SKBR3 | BT20 | T47D | HCC1569 | HCC1143 | |
| PTP4A3 | | | | | | | | | HMEC | BT20 | T47D | SKBR3 | MCF7 | HCC1143 | MDAMB157 | HCC1569 |
| RAD21 | | | | | | | | | HMEC | BT20 | SUM149 | T47D | HCC38 | CAL51 | HCC1954 | |
| S100A9 | | | CAL51 | T47D | HCC38 | SUM149 | BT20 | HCC1954 | HMEC | | | | | | | |
| SEC61G | | | HCC1569 | SKBR3 | T47D | MDAMB157 | HCC1143 | MCF7 | HMEC | BT20 | | | | | | |
| SPINT2 | | MDAMB436 | MDAMB157 | SUM149 | HCC1569 | CAL51 | HS578T | HCC38 | HMEC | HCC1143 | MCF7 | | | | | |
| TTC35 | | | | | | | | SKBR3 | HMEC | BT20 | MDAMB231 | CAL51 | T47D | HCC1143 | | |
| TTK | | | | | | BT474 | SKBR3 | HCC38 | HMEC | MDAMB231 | CAL51 | MDAMB436 | | | | |
| UBE2T | | | | | | | | | HMEC | MDAMB157 | SKBR3 | MCF7 | BT20 | HCC1569 | T47D | HCC1143 |
| VPS45 | | | | | | | SUM149 | HCC1143 | HMEC | CAL51 | MCF7 | MDAMB436 | HCC1187 | | | |

Supplementary Fig. 4 Patel *et al.*,

**Supplementary Figure 4 Analysis of association between gene expression level compared to non-malignant HMEC and effect on NPI upon knockdown of validated hits.**

Cell lines ranked according to gene expression level (mRNA) centered to the non-malignant cell line HMEC. Red represents cell lines in which knockdown of that gene produced an effect on growth inhibition and blue represents cell lines in which knockdown of target gene produced no effect on growth inhibition.

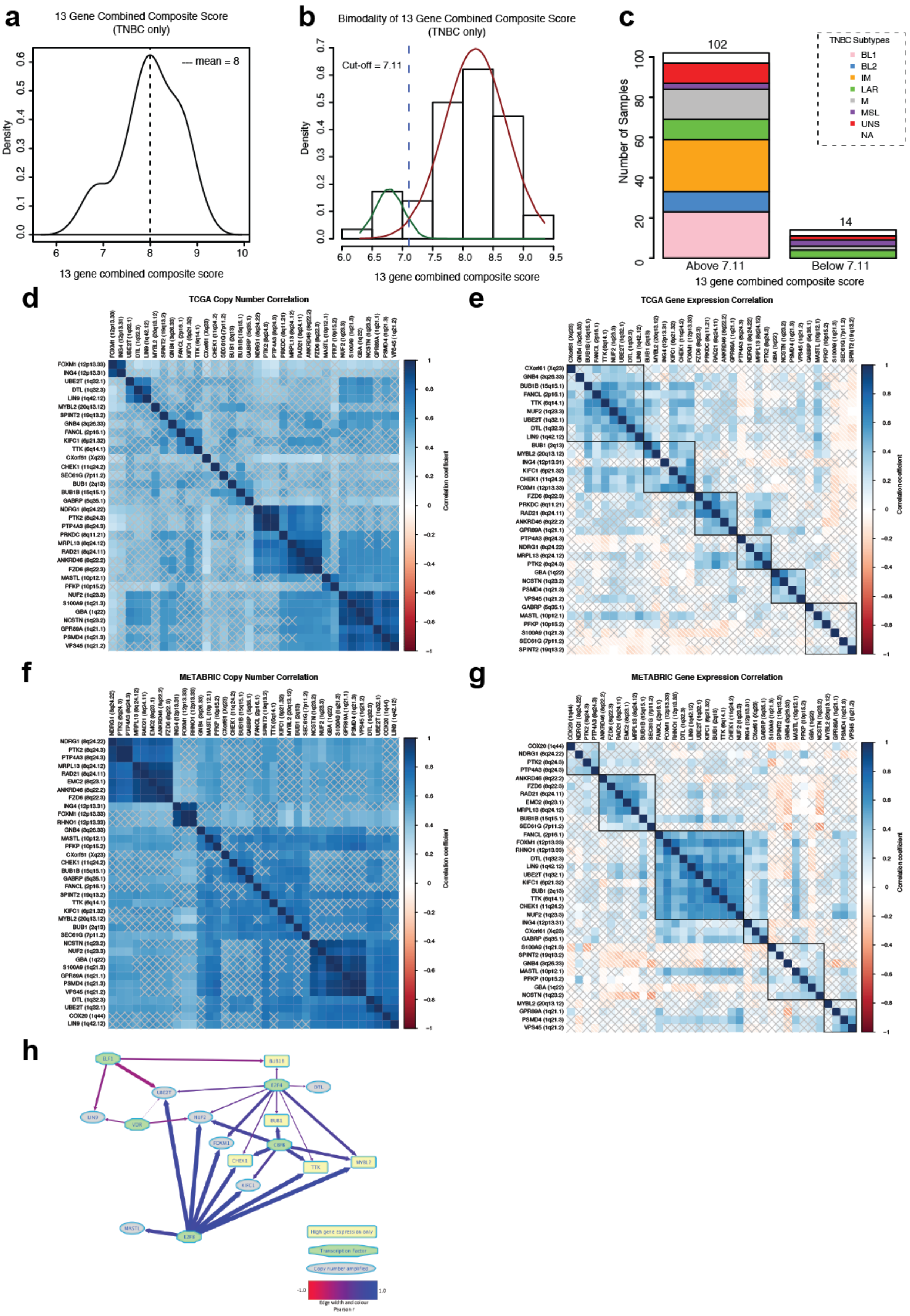


Supplementary Fig. 5 Patel *et al.,*

**Supplementary Figure 5 Copy number and gene expression correlation of validated tumor addiction genes.**

**a.** Distribution of a gene signature-score, established by a weighted summarization of the expression levels from the 13 genes, in TNBCs from the Guy's TNBC enriched cohort, dashed line indicates the mean score (n=116). **b.** Bimodality of the 13 gene signature-score on TNBCs from the Guy's TNBC enriched cohort (n=116). Dashed line indicates the cross-over of the 2 populations at 7.11. **c.** Comparison of TNBC subtype of TNBC tumors above and below the bimodal cut-off of 7.11 from Guy's TNBC enriched cohort (n=116). **d and e.** Gene copy number and expression correlation heatmap of 37 genes only in the TCGA TNBC (n=97), same as in figure 2. **f and g.** Gene copy number and expression correlation heatmap of 37 genes only in the METABRIC TNBC (n=101), same as in figure 2. **h.** Common transcription factor network among the 13 genes involved in mitosis. iRegulon analysis using 750 ChIP-seq data (ENCODE uniform signals) on 500bp sequences upstream of the transcriptional start sites for each of the 13 genes. The top 5 transcription factors were selected based on descending NES scores, and an interaction network was created including the 13 genes. The thickness of the network edges illustrates the Pearson's correlation values derived from expression profiles correlation in the Guy's TNBC enriched cohort.

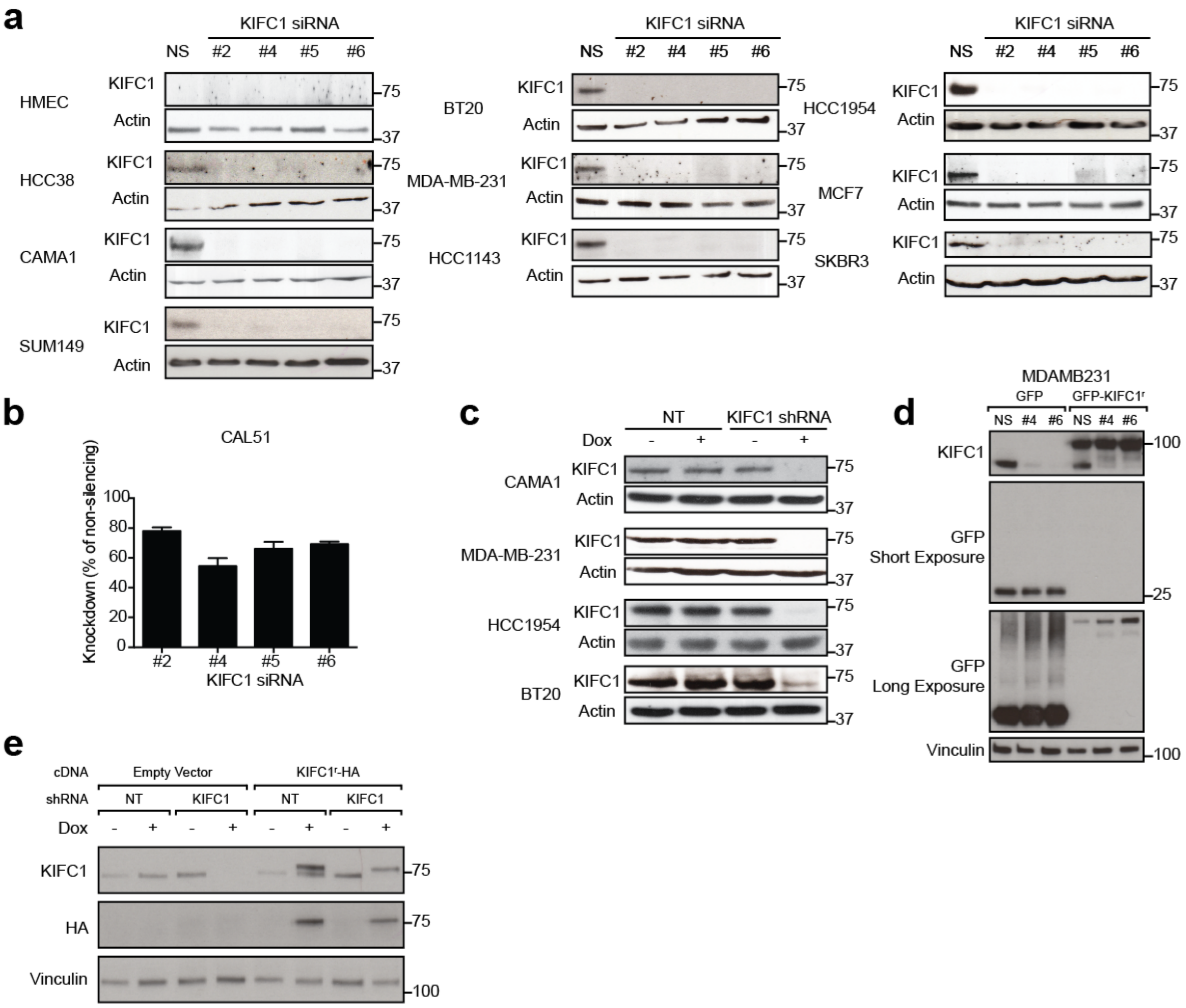


Supplementary Fig. 6 Patel *et al.,*

**Supplementary Figure 6 Confirmation of KIFC1 knockdown.**

**a.** KIFC1 and β-actin western blot of HMEC, HCC38, CAMA1, SUM149, BT20, MDA-MB-231, HCC1143, MCF7, HCC1954 and SKBR3 with non-silencing (NS) and KIFC1 siRNA #2, #4, #5 and #6. **b.** Graph showing mean percentage knockdown of KIFC1 by qPCR compared to non-silencing control for KIFC1 siRNA #2, #4, #5 and #6 in CAL51. Error bars represent the SEM, n=3. **c.** KIFC1 and β-actin western blot of CAMA1, MDA-MB-231, HCC1954 and BT20 with non-targeting (NT) or KIFC1 shRNA with or without doxycycline (dox) as indicated in panel. **d.** KIFC1, GFP and

vinculin western blot of MDA-MB-231 GFP alone and MDA-MB-231 GFP-KIFC1$^r$ with NS and KIFC1 siRNA #4 and #6. **e.** KIFC1, HA and vinculin western blot of MDA-MB-231 with inducible NT shRNA and either inducible empty vector or inducible KIFC1$^r$-HA and MDA-MB-231 with inducible KIFC1 shRNA and either inducible empty vector or inducible KIFC1$^r$-HA. Cells were treated with or without dox as indicated in panel.

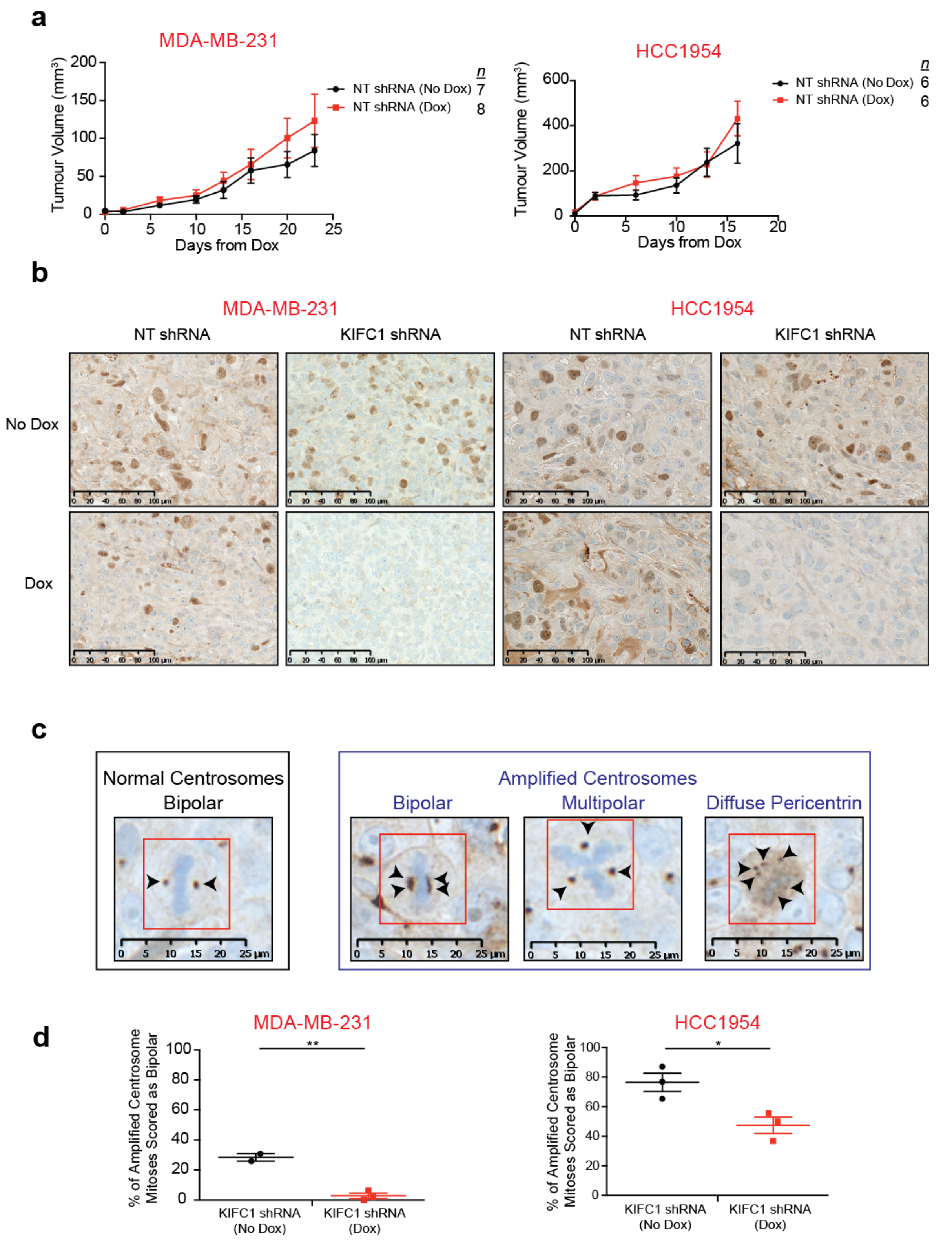


Supplementary Fig. 7 Patel *et al.*,

**Supplementary Figure 7 Non-targeting shRNA cell line xenograft growth *in vivo*.**

**a.** Nude hosts were orthotopically injected with MDA-MB-231 or HCC1954 with inducible non-targeting (NT) shRNA and were treated with (red) or without (black) doxycycline. Data points represent the mean and error bars represent the SEM from two independent experiments. Two-way ANOVA with Sidak's multiple comparisons test ($p>0.05$). **b.** Representative images of KIFC1 IHC staining of MDA-MB-231 and HCC1954, NT and KIFC1 shRNA xenografts treated with or without dox as indicated in panel. Scale bar is 100μm. **c.** Representative images of mitotic cells in MDA-MB-231 KIFC1 shRNA xenografts. Red box shows mitotic cell and arrows indicate centrosomes. Scale bar is 25μm. **d.** Histologic analysis of mitoses in MDA-MB-231 and HCC1954 cells with inducible KIFC1 shRNA treated with (red) or without (black) doxycycline. Sections were stained with pericentrin to visualize centrosomes; the mitoses were categorized into cells with either normal or amplified centrosomes. The mitoses in cells with amplified centrosomes were sub-categorized into those with bipolar, multipolar or diffuse pericentrin staining with absence of mitotic polarity. Mean percentage of cells with amplified centrosomes that were capable of bipolar mitosis 4 days after start of treatment is shown, error bars represent the SEM, n=3. Students T-test: * $p<0.05$, ** $p<0.01$.

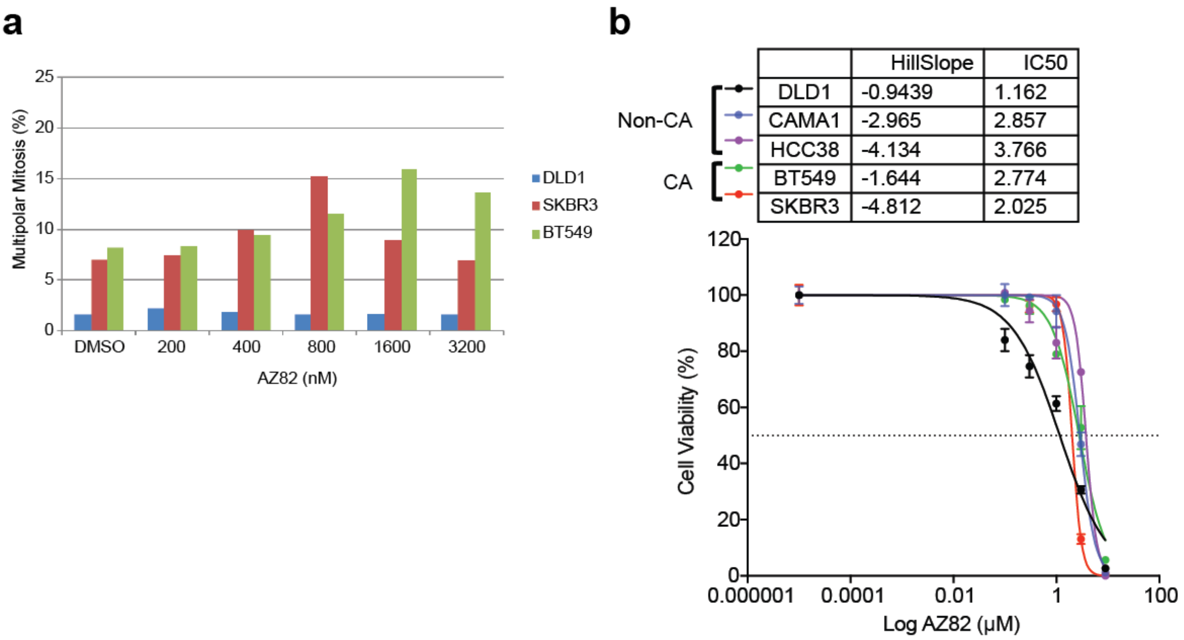


Supplementary Fig. 8 Patel *et al.,*

**Supplementary Figure 8 Treatment of centrosome amplified and non-centrosome amplified cell lines with KIFC1 inhibitor AZ82.**

**a.** Cells were treated with proTAME, an APC/C inhibitor, for 3h to enrich for mitotic population and then treated with the indicated concentrations of AZ82 for another 3h. The cells were fixed in methanol and stained with Aurora A, to identify spindle poles and phospho-Histone H3 to identify mitotic cells. Images were acquired on the Operetta platform and automated analysis of mitotic poles/cell was performed with Harmony software. Percent multipolar mitoses are shown in relation to bipolar mitoses. DLD1 cells are colon adenocarcinoma cells with no centrosome amplification; SKBR3 and BT549 are breast cancer cells with centrosome amplification. At least 600 mitoses/condition were quantified. **b.** Cell viability effect of AZ82 in non-centrosome amplified (DLD1, CAMA1, HCC38) versus centrosome amplified (BT549, SKBR3) cell lines, as determined by SRB assay. Cells were

seeded in 48-well plates at a number optimized for each cell line. Next day, three-fold dilutions of the AZ82 were made in culture medium so that, when diluted 2X, the final concentration in the wells was from 9 μM to 0.1 μM (5 concentrations). For control, DMSO was added. The cells were incubated for a period corresponding to 5 population doubling times for each cell line. On the last day, cells were fixed and the number of viable cells determined by SRB assay. The mean SRB absorbance readings were analysed using Graphpad PRISM to measure the $IC_{50}$ of AZ82, error bars represent the SEM, n=3. Non-CA: non-centrosome amplified; CA: centrosome amplified.

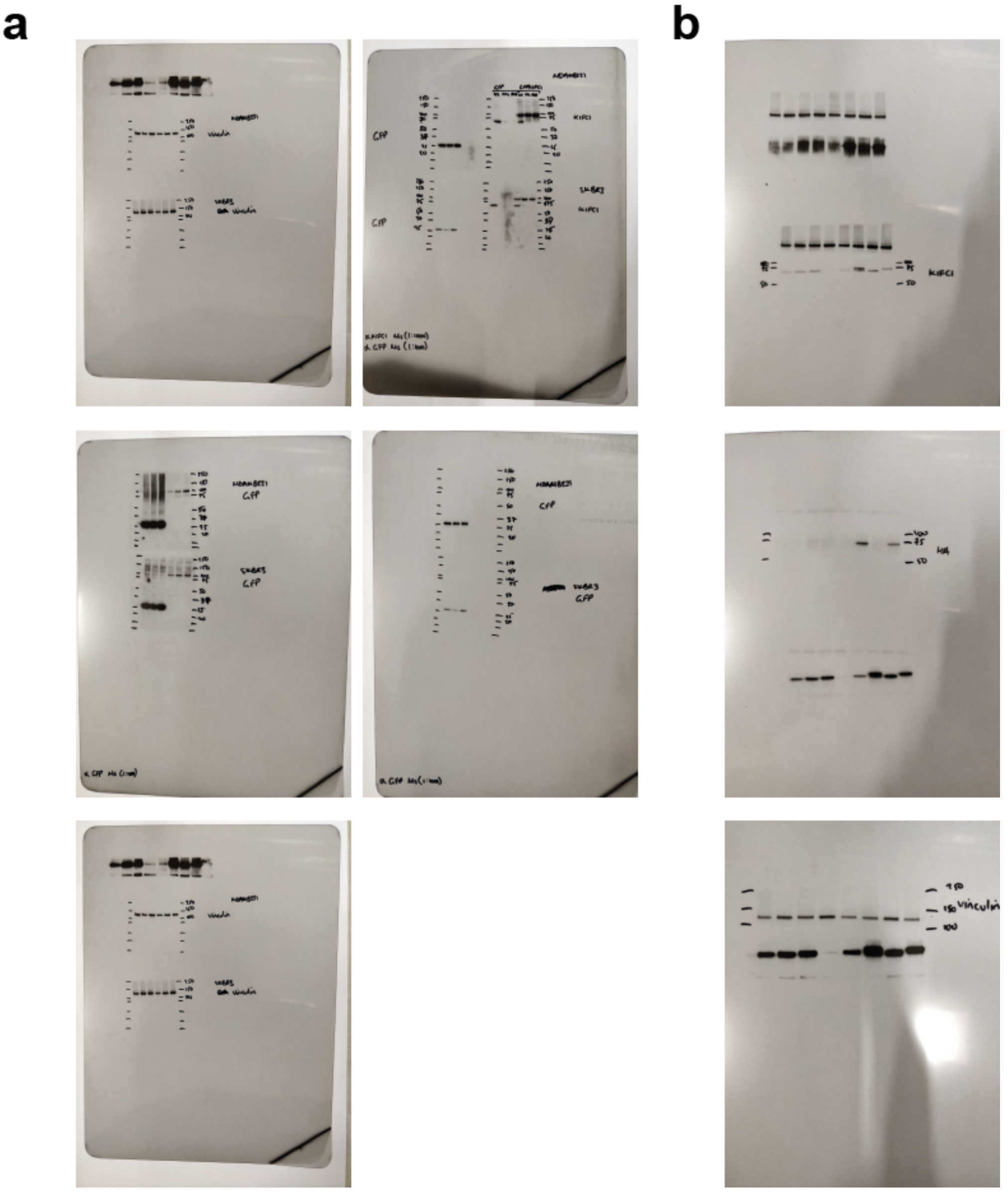

**Supplementary Figure 9 Full scans of western blots.**

**a.** Full scans of western blots shown in Supplementary figure 6d. **b.** Full scans of western blots shown in Supplementary figure 6e.
