# Supplementary material for: Integrated genomics and functional validation identifies malignant cell specific dependencies in triple negative breast cancer
Source: Nat Commun. 2018 Mar 13;9:1044. doi: 10.1038/s41467-018-03283-z (PMC5849766; doi:10.1038/s41467-018-03283-z)
Supplement: Supplementary file 2 — Description of Additional Supplementary Files [file 41467_2018_3283_MOESM2_ESM.pdf]

Description of Supplementary Files

File Name: Supplementary Data 1
Description: **Clinico-pathologic information of cohort of TNBC enriched primary breast cancers.** Table showing information on patient breast samples such as sample type, ER, PR and HER2 status of breast tumors, histological grade and recurrence.

File Name: Supplementary Data 2
Description: **Target ID data platform.** Table showing the parameters included in the Target ID weighted scoring system separated into 5 blocks. The limits and thresholds of each parameter are also shown.

File Name: Supplementary Data 3
Description: **Target ID score for each gene.** Table showing Gene ID, final score and breakdown of this score for each block as described in Supplementary Data 2.

File Name: Supplementary Data 4
Description: **Selected genes and hit lists.** Table showing genes identified through both the copy number dependent gene expression analysis, the gene expression centered analysis and genes common to both of these approaches. The genes identified as 'Top 10' and the genes that passed the validation step for these are shown. The table also presents the 120 genes taken forward for the two stage analysis and the positive hits for both stages of this analysis.

File Name: Supplementary Data 5
Description: **Candidate genes interrogated in METABRIC and TCGA TNBCs.** Table showing comparison of gene expression and copy number amplification of the 130 candidate genes in Guy's TNBC enriched cohort, METABRIC and TCGA BRCA.

File Name: Supplementary Data 6
Description: **Gene lots and cell line assignments.** Table showing the genes grouped into lots and the cell lines that each lot was tested on. Breast cancer subtype of cell lines are indicted with colored legend on the right. In green, non-malignant cell lines. In blue, cell lines that are ER/PR+. In purple, cell lines that are HER2+. In red, cell lines that are ER/PR/HER2- (TNBC).

File Name: Supplementary Data 7
Description: **RNAi based validation of Top 10 genes.** Table showing the siRNA knockdown efficiency of 3 independent siRNAs on each of these genes in HCC1143. Highlighted in red are siRNA species that did not knockdown the gene to a sufficient level to allow be included in the analysis (>70%). Table below shows the NPI of each siRNA on each cell line for each gene. Highlighted in red are the NPI values for the gene and cell line where the siRNA was deemed to not have silenced the gene to a sufficient level (>70%).

File Name: Supplementary Data 8
Description: **Primary functional validation using pooled-RNAi based approach.** Table showing mean NPI value for each gene in each cell line. The genes are grouped into lots as described in Supplementary Data 5.

File Name: Supplementary Data 9
Description: **Secondary functional validation after deconvolution of the pool of siRNA.** Table showing mean NPI values and gene knockdown for the same cell line for each gene. Genes with insufficient data for other reasons are excluded from the table. Ø – Equivocal evidence of knockdown.

File Name: Supplementary Data 10
Description: **Quality control for primary functional validation.** Table showing overall mean R squared value for all replicates within the analysis of each cell line in each lot.

File Name: Supplementary Data 11
Description: **Comparison of validated hits from Guy's TNBC enriched cohort and results from COLT-Cancer study.** Table showing cell line by cell line comparison between mean NPI values of validated hits from primary validation from Guy's TNBC enriched cohort with zGARP scores of breast cancer cell lines from the COLT-Cancer study.

File Name: Supplementary Data 12
Description: **Antibodies used in this study.** Table showing antibodies, source Ig, source company, catalogue number, working dilution and use. IF – immunofluorescence, WB – western blotting, and IHC – immunohistochemistry.

File Name: Supplementary Movie 1
Description: **Normal bipolar mitosis.** Time-lapse video of MDA-MB-231 cells with inducible NT shRNA and doxycycline going through a normal bipolar mitosis. Video has been cropped and the brightness and contrast levels and tonal range have been adjusted for visualization of cell of interest.

File Name: Supplementary Movie 2
Description: **Catastrophic multipolar mitosis.** Time-lapse video of MDA-MB-231 cells with inducible KIFC1 shRNA and doxycycline going through a prolonged mitosis and enters a multipolar configuration followed by mitotic catastrophe. Video has been cropped and the brightness and contrast levels and tonal range have been adjusted for visualization of cell of interest.

File Name: Supplementary Movie 3
Description: **Multipolar mitosis and failed cytokinesis.** Time-lapse video of MDA-MB-231 cells with inducible KIFC1 shRNA and doxycycline going through a prolonged mitosis and enters a multipolar configuration followed by anaphase but failing cytokinesis resulting in a multinucleated cell.
